# Virally mediated expression of a biologically active peptide to restrain the nuclear functions of ERK1/2 attenuates learning extinction but not acquisition

**DOI:** 10.1186/s13041-025-01190-1

**Published:** 2025-03-14

**Authors:** Bar Izkovich, Adonis Yiannakas, Sapir Ne’eman, Sailendrakumar Kolatt Chandran, Kobi Rosenblum, Efrat Edry

**Affiliations:** 1 Sagol Department of Neuroscience, University of Haifa, Haifa, Israel; 2 European University of Cyprus Medical School, Frankfurt, Germany; 3 Center for Gene Manipulation in the Brain, University of Haifa, Haifa, Israel

## Abstract

Peptide drug technologies offer powerful approaches to develop potent and selective lead molecules for therapeutic and research applications. However, new and optimized delivery approaches are necessary to overcome current pitfalls including fast degradation in cells and tissue. Extracellular signal-regulated kinases 1/2 (ERK1/2) exemplifies proteins that play crucial and varied roles within distinct cellular compartments. Here, we established an innovative method, based on viral vectors, which utilizes the endogenous biogenesis of neurotrophins to deliver and express a biologically active peptide to attenuate specifically ERK1/2 nuclear functions in specific brain area of the adult forebrain. In contrast to our hypothesis, nuclear functions of ERK1/2 in the forebrain are fundamental for the extinction of associative-aversive memories, but not for acquisition, nor for retrieval of these memories. Our research demonstrates the feasibility and applicability of viral vectors to deliver a peptide of interest to manipulate specific molecular processes and/or protein interactions in specific tissue.

**Supplementary Information:**

The online version contains supplementary material available at 10.1186/s13041-025-01190-1.

## Introduction

Peptide-based inhibitors represent a class of promising candidates for both basic research and therapeutic applications due to their resemblance to endogenous ligands, high specificity, and low toxicity. Unlike traditional small molecules, peptide inhibitors can target specific modalities within multifunctional proteins involved in complex signaling cascades [1]. However, small molecule-based drugs continue to dominate drug research due to their low production costs, oral administration options, and membrane permeability [2, 3]. Despite these benefits, peptides face inherent challenges related to stability and membrane permeability [1].

In the brain, naturally occurring neuropeptides are released by neurons, and often function in a paracrine manner, influencing neighboring cells [4]. Most growth factors and hormones are synthesized as pre-pro-proteins, which are processed to the biologically active mature protein. The pre- and pro-domains are cleaved from the precursor protein in the secretory pathway or, in some cases, extracellularly, to generate an active peptide [5]. Here, we aimed to generate a recombinant preproPEPTIDE precursor that may undergo equivalent biogenesis within virally-transduced cells to generate a biologically active peptide.

The mitogen-activated protein kinase (MAPK) signaling cascade is a crucial pathway regulating multiple cellular processes in all cells, including neurons, ranging from cell proliferation and differentiation, to brain development, along with learning and memory [4]. Disruption of different elements in this signaling pathway results in cognitive impairment [6]. Within this pathway, the serine/threonine kinases ERK1/2 play a pivotal role by phosphorylating substrates both in the cytosol and nucleus [7]. ERK1/2 normally resides in the cytoplasm but can rapidly translocate to the nucleus following phosphorylation-activation. Upon activation, ERK1/2 translocates to the nucleus by virtue of its nuclear translocation signal (NTS), that allows binding to importin7, facilitating nuclear entry [8]. Once in the nucleus, ERK1/2 mainly functions as gene transcription regulator by modifying the function of transcription factors (TFs) and histones [9]. Notably, ERK-dependent induction of PSD-95 in the gustatory cortex has been shown to be necessary for taste learning, highlighting ERK1/2’s involvement in memory processes related to taste [10]. Peptide-based inhibitors can be designed to mimic such and disrupt such protein interactions, offering potent and selective control of signaling pathways. The EPE peptide, a phosphomimetic peptide, attenuates the nuclear translocation of ERK1/2 by disrupting the binding of phosphorylated its NTS with importin7, and preventing nuclear translocation [11].

In this study, we designed viral vectors to express peptide precursors that take advantage of the endogenous biogenesis of neurotrophins, such as brain-derived neurotrophic factor (BDNF) and nerve growth factor (NGF). These vectors harbor the BDNF/NGF pre-pro domains encoding sequences. However, the mature BDNF/NGF domains were replaced with the sequence of the EPE peptide, previously shown to inhibit ERK1/2 nuclear translocation (LV: proBDNF-EPE; proNGF-EPE) [11, 12]. Importantly, these constructs did not produce mature BDNF or NGF but instead retained only the pro-domain, fused to the EPE peptide sequence. This design ensured that the observed effects were due to inhibition of ERK nuclear translocation rather than neurotrophic factor activity. The approach resulted in the generation of functional viral vectors that expressed engineered precursors, targeting translocation of ERK1/2 to the nucleus. Viral mediated delivery of the EPE peptide in COS7 (monkey kidney fibroblasts), decreased nuclear/cytosolic ERK1/2 ratio, and reduced phosphorylation of Elk1, a key nuclear target typically activated by ERK1/2 within the nucleus [13, 14]. Delivering LV proBDNF-EPE to the CA1 region of the mouse hippocampus impaired the extinction of contextual fear memories without affecting their acquisition and retrieval. Importantly, LV proBDNF-EPE manipulation at the CA1 region suppressed phosphorylation of its nuclear targets Elk1 and mitogen- and stress-activated kinase 1 (MSK1). Activation of ERK1/2 signaling is critical for fear memory consolidation, as it drives synaptic plasticity and long-term potentiation (LTP) by phosphorylating key transcription factors, which regulate gene expression necessary for stabilizing memory traces and modulating the balance between consolidation and extinction [15–17]. Since Elk1 and MSK1 are critical mediators of ERK-driven transcriptional programs linked to synaptic plasticity and memory formation, their suppression following EPE expression suggests a disruption in nuclear ERK-dependent gene expression required for extinction learning [18, 19]. To assess the generalization of the requirement for ERK1/2 nuclear functions in extinction learning, we injected LV proBDNF-EPE into the anterior insular cortex (aIC) before conditioned taste aversion (CTA) learning and measured again clear deficits in extinction learning, but not in memory acquisition, nor in retrieval. ERK1/2 signaling is recognized as an instrumental component of the molecular machinery underlying valence-specific taste memory encoding and consolidation in the IC neurons [20–22]. Our results suggest that localized expression of EPE peptide through viral vector delivery [23], can selectively inhibit a particular modality of MAPK signaling—namely, nuclear ERK1/2 activity—thereby modulating the molecular mechanisms underlying distinct components of learning [24]. Moreover, the results provide solid proof of concept for a versatile method that enables the fine manipulation of protein functions using peptide inhibitors. This strategy overcomes key limitations of traditional peptide-based drugs, such as poor stability and limited membrane permeability, by leveraging an endogenous biogenesis and protective processing pathways of neurotrophins.

## Materials and methods

### Cell lines

COS7 and HEK293FT cell lines were purchased from ATCC and Thermo Fisher Scientific, respectively, and were cultured in Dulbecco’s modified Eagle medium (DMEM) with 10% fetal bovine serum (Gibco), penicillin (100 IU/ml), streptomycin (100 mg/ml), and L-glutamine (2mM) and kept in a 37 °C incubator with 5% CO_2_. Cells were maintained up to 80–90% confluence and passaged every 2–3 days.

### Animals

Animals used were 8-to 12-week-old wild type (WT) adult (C57BL/6) male mice. Mice were kept in the local animal resource unit at the University of Haifa in an environment that is temperature-controlled and under a 12 h dark/light cycle. Water and chow pellets were available ad libitum. All experiments and procedures conducted were approved by the University of Haifa Animal Care and Use Committee under ethical license 525/17, in accordance with the National Institutes of Health guidelines for the ethical treatment of animals.

### Peptide

The synthetic EPE peptide (GQLNHILGILGEPEQED) was conjugated in its N-terminal to a modified TAT (YARAAARQARA) and HA tag (YPYDVPDYA) sequences. EPE peptide was purchased from Syntezza Bioscience. The peptide was > 90% pure and kept as 10mM dimethylsulphoxide (DMSO) stock solution at − 20 °C. All concentrations and time points were calibrated prior to the experiments.

### Recombinant lenti-viral vector production

Transfer plasmids expressing the proBDNF-EPE and proNGF-EPE constructs, as well as a control vectors expressing the pro-domain of BDNF fused to control peptide sequence (proBDNF con) or GFP, were generated using conventional cloning techniques. Self-inactivating, third-generation HIV-1-based viral vectors were produced by transient co-transfection of four endotoxin-free plasmids (Endo Free Plasmid Maxi Kit, catalog #12362, Qiagen) in Invitrogen Human Embryonic Kidney 293FT (HEK293FT; Thermo Fisher Scientific) cells [25]. Cells were cultured in Invitrogen DMEM with 10% fetal bovine serum (Thermo Fisher Scientific), penicillin (100 IU/ml), and streptomycin (100 mg/ml), and were kept in a 37 °C incubator with 5% CO_2_. Transfection was performed using PEI (SignaGen), according to manufacturer instructions. Following a 17 h incubation, the medium was replaced with DMEM supplemented with 10% fetal bovine serum. At 48 h post-transfection, the medium was harvested, cleared by low-speed centrifugation (800 RCF, 10 min, 4 °C), and filtered using 0.45 μm pore filters (Nunc). Vectors were then concentrated by ultracentrifugation using the SW28 rotor (Beckman Coulter; 19,000 rpm, 2.5 h, 15 °C), and the pellets obtained were finally suspended in HBSS (Sigma-Aldrich), aliquots were prepared and stored at − 80 °C. Vectors were titrated by transduction of HEK293FT cells using serial dilutions of the viral vector stock, along with 8 μg/μl Polybrene (Sigma-Aldrich), and GFP expression was analyzed by flow cytometry analysis 2 days later. The lentiviral titer obtained was 10^8^ transducing units/ml.

### In vitro cell transduction

Titrated vectors were used for COS7 in vitro cell transduction (MOI 5). Briefly, COS7 cell line were seeded in 24 well plates (100,000 cells/well), in 250 μl of medium containing 8 μg/ml Polybrene (Sigma-Aldrich). Cells were transduced with LV-proBDNF/proNGF-EPE or control vector and incubated for 24 h. On the next day, the cells were supplemented with fresh medium (250 μl), were grown up to 80–90% confluence, and then passaged to a 6-well plate for experimental procedures.

### Nuclear/cytosolic fractionation

The Nuclear/Cytosolic Fractionation Kit (Cell Biolabs) was used for the isolation of nuclear extract from the cytoplasmic fraction of mammalian cells. Cells were cultured to 80–90% confluence. The medium was aspirated, and cells were washed twice with warm PBS. Then, the cells were detached from the plates by scraping, collected into an appropriate conical centrifuge tube with 2.5 ml of PBS, and centrifuged for 5 min (600xg, 4 °C). The pellet was subsequently treated according to manufacturer’s instructions.

### Sample preparation and brain dissection

Lysis of COS7 cells was done by RIPA buffer (Sigma-Aldrich, Israel) supplemented with phosphatase inhibitor (Sigma-Aldrich, Israel) and protease inhibitor mixtures (Sigma-Aldrich, Israel). All the work was done on ice to prevent degradation of proteins. Mice were sacrificed by cervical dislocation, and brains were immediately removed, and flash frozen on a tin foil platform floated on liquid nitrogen. Brains were kept in − 80 °C, and eventually were transferred to a Leica CM 1950 Cryostat and equilibrated to − 15 °C. Four 500 μm thick coronal sections were sliced slowly, and hippocampal CA1 regions from both hemispheres were collected by a mouse tissue puncher. Brain tissues were homogenized in 50 μl of ice-cold homogenization buffer (HEPES 10mM pH 7.4, EDTA 2mM pH 7.4, EGTA 2mM pH 7.4, DTT 0.5mM, all from Sigma-Aldrich, Israel), 1X protease inhibitor mixture (Sigma-Aldrich, Israel); and 1X phosphatase inhibitor mixture (Sigma-Aldrich, Israel).

### SDS-page and western blotting

Protein samples in SDS sample buffer were subjected to SDS-PAGE (7.5–10%) and western blot analysis. All Lanes were loaded with 5 μg of protein. Following transferring into a nitrocellulose or PVDF membranes using Trans-Blot® TurboTM Transfer System (Bio-Rad), membranes were blocked in 1% BSA with 0.02% Na-Azide for 1 h at room temperature (RT). Next, membranes were incubated overnight at 4 °C with relevant primary antibodies: p44/42 MAPK (Erk1/2) (1:1000, Cell Signaling) and Phospho-P44/42 MAPK (T202/Y204) (1:1000, Cell Signaling); Elk-1 (1:1000, Cell Signaling) and phosph-Elk-1 (S383) (1:1000, Cell Signaling); MSK1 (C27B2) (1:1000, Cell Signaling) and phospho-MSK1 (T581) (1:1000, Cell Signaling); Anti-Glutamate Receptor 2 (1:1000, Millipore) and β-Actin (1:1000, Abcam). On the following day, three 5-min washing steps were performed in Tris-buffered saline (140 mM NaCl, 20 mM Tris, pH 7.6) supplemented with 0.1% Tween 20 (TBS-T), and then membranes were incubated for 1 h at RT with the relevant secondary HRP-linked antibody: Goat-anti-Rabbit (IgG) HRP conjugated; Goat-anti-Mouse (IgG) HRP conjugated (1: 10,000, Jackson ImmunoResearch). Immunodetection was accomplished with the WESTAR SUPERNOVA Chemiluminescence substrate (CYANAGEN), and images were acquired and analyzed using AI600 Imager. Each sample was measured relative to the background and quantified relative to a control sample.

### Immunofluorescence

Virally transduced COS7 cells were seeded (75,000 cells/well) in a 12-well plate containing glass cover slips coated with Poly-L-lysine (0.001%, Sigma Aldrich) and were grown to 50–70% confluence. Cells were then fixed in cold 4% formaldehyde solution in phosphate saline buffer (PBS) for 20 min, washed, and permeabilized with 0.5% Triton X-100 in PBS. Cells were then incubated for 1 h at RT in blocking solution (Triton X-100 0.5%, 10% FBS, 0.3% BSA, in PBS) and subsequently incubated for 2 h at RT with the primary antibody, anti- Ha-tag (1:1000, Cell Signaling) diluted in the same blocking solution. After three washing steps (0.1% Triton in PBS), cells were incubated for an additional 1 h at RT with the corresponding secondary antibody: Rhodamine RedTM goat anti-Rabbit IgG, diluted to a final concentration of 1:500 in saturation buffer. Cells were washed and coverslips were mounted on FluoroshieldTM with DAPI (Sigma Aldrich). Images were acquired using an Olympus IX81 microscope with Hamamatsu ORCA R2 camera controlled by cellSens Dimension software (version 1.16). Equivalent exposure conditions were used for all slides and images.

### Surgeries and microinjection of viral vectors

Mice were restrained in a stereotactic apparatus (KOPF, USA), anesthetized with 4% Isoflurane (USP 100%, Terrell), and maintained at 1.5% isoflurane during the surgery. Microinjection of viral vectors (10^8^ TU/Ml) was performed by directly injecting 1 μl (0.1 μl/min) to the Insula (coordinates relative to bregma: AP: +0.86, LM: ±3.40, DV: -3.80) or to the hippocampal CA1 region (coordinates relative to bregma: AP: -1.78, LM: ±1.20, DV: -1.63), as previously described [26, 27]. The injection was performed using a 10 μl microsyringe (#7653-01, HAMILTON). Following the surgery, mice were treated with an analgesic (dipyrone 50%, intramuscular) and topical antibiotic (synthomycin 5%), and allowed to recuperate for at least one week before the behavioral test.

### Contextual fear conditioning

On the training day, mice were transferred to a nearby testing room for habituation next to the conditioning chambers as previously described [28]. Each chamber was wiped with 70% ethanol solution before training, and each mouse was placed in the chamber individually during training. Each mouse was placed in context A (with light [20-W bulb] and a 16-bar metal grid floor) and received pairings between a tone (2.9 kHz, 30 s at 80 dB, conditioned stimuli; CS) and a foot shock (0.5-mA for 2 s) that co-terminated with the tone (unconditioned stimuli; US). After the shock presentation, an inter-trial interval (60 s) precedes a second identical trial. Chambers were cleaned with 70% ethanol followed by double distilled water (DDW) between successive sets of mice. Assessment of the conditioned fear of the training context (context A) was conducted 24 h post conditioning. Mice were placed in the conditioning chamber (context A) for 5 min without a tone or a foot shock. The same test was done in the six subsequent days to evaluate extinction rates. The movement of the mice in the fear-conditioning chamber was recorded and analyzed by Freeze Frame 3.0 software (Coulbourn Instruments, PA, USA). The freezing score was calculated as the percentage of time for which the mice remained immobile.

### Conditioned taste aversion

Following the injection of the viral vectors (GFP or proNGF/proBDNF-EPE) to the insular cortex, animals were allowed to recover for 7 days. Animals were trained to drink from pipettes (days 1–5): On day 1 they went through water deprivation, and on the next two days they were allowed to drink from one pipette containing 5 ml of water. On the next two days, mice were presented with the pipette for 20 min per day. Conditioning was performed on day 6: Mice were presented with a saccharin (0.5%) containing pipette (1 ml) for 20 min. Forty minutes later; they were injected with 1.5% body weight of LiCl (0.075 M). On days 7–8, mice were presented with water (5 ml) for 20 min per day. The test was performed in an extinction mode: on days 9–15, mice were presented simultaneously with two pipettes, one containing 5 ml of saccharin and one containing 5 ml of water, for 20 min each day. The multiple choices of water and saccharin during the test were to minimize the effect of arbitrary choice of a saccharin pipette by the thirsty mice. The aversion index was defined as [ml water/ (ml water + ml saccharin) x100] consumed during the test, i.e., 50% defined as chance level, and the higher the aversion index, the more the mice prefer water compared to saccharin. The aversion index was thus defined to minimize the effect of individual variability in fluid consumption on the results. On days 16–18, mice were presented with one pipette of 2 ml saccharin (0.5%) for 20 min each day. On the following day, mice were injected with 1.5% body weight of LiCl (0.075 M) 40 min prior to being provided with 2 ml of water (unpaired), for CTA reinstatement. Twenty-four hours later, mice were subjected to a choice test between saccharin and water, 20 min prior to deep anesthesia and cardia re-perfusion using 4% paraformaldehyde.

### Electrophysiological recordings

*Brain Slice Electrophysiology and Whole-cell Patch Clamp.* The slice electrophysiology and recording parameters were used as described previously [29, 30]. Briefly, the mice (8–10 weeks) were deeply anesthetized using isoflurane, while brains were extracted following decapitation. Three-hundred um thick coronal brain slices obtained with a Campden-1000® Vibratome. Slices were cut in an ice-cold sucrose-based cutting solution containing the following (in mM): 110 sucrose, 60 NaCl, 3 KCl, 1.25 NaH2PO4, 28 NaHCO3, 0.5 CaCl2, 7 MgCl2, 5 D-glucose, and 0.6 ascorbate. The slices were allowed to recover for 30 min at 37 °C in artificial CSF (ACSF) containing the following (in mM): 125 NaCl, 2.5 KCl, 1.25 NaH2PO4, 25 NaHCO3, 25 D-glucose, 2 CaCl2, and 1 MgCl2. Slices will be then kept for an additional 30 min in ACSF at room temperature until electrophysiological recording. The solutions were constantly aerated with carbogen (95% O2, 5% CO2).

*Intracellular whole-cell recordings.* After the recovery period, slices were placed in the recording chamber and maintained at 32–34 °C with continuous perfusion of carbogenated ACSF (2 ml/min). Brain slices containing the CA1 were illuminated with infrared light and pyramidal cells visualized under a differential interference contrast microscope with 10X or 40X water-immersion objectives mounted on a fixed-stage microscope (BX51-WI; Olympus®). The image was displayed on a video monitor using a charge-coupled device (CCD) camera (Orca Retiga 2®, Hamamatsu Japan). The recordings were made from the soma of CA1 pyramidal cells identified by green florescence. Liquid junction potential (10 mV) was not corrected online. Pipette capacitance and series resistance were compensated and only cells with series resistance smaller than 20 MΩ were included in the dataset. To record mEPSCs, the recording electrode was pulled from a borosilicate glass pipette (3–5 M) using an electrode puller (P-1000; Sutter Instruments®) and filled with a K-gluconate-based internal solution containing the following (in mM): 130 K- gluconate, 5 KCl, 10 HEPES, 2.5 MgCl2, 0.6 EGTA, 4 Mg-ATP, 0.4 Na3GTP and 10 phosphocreatine (Na salt). The osmolarity was 290 mOsm, and pH of 7.3. mEPSCs were recorded in voltage clamp mode at a holding potential of − 70 mV. 50μM bicuculline (Tocris) and tetrodotoxin (TTX − 1 μM (Tocris) were added to the external ACSF solution. Data were acquired by Double IPA® Integrated Patch Clamp Amplifiers with Data Acquisition System (Sutter Instruments®). Data sampled at 20 kHz and filtered at 2 kHz. Series resistance, Rin, and membrane capacitance were monitored throughout, and experiments where resistance changed > 20%, were discarded. Data quantification was done with Sutter Patch (Version 2.2, Sutter Instruments, Novato, CA) and subsequently analyzed using GraphPad Prism®.

### Statistical analysis

Mice were assessed and data were processed by investigators blinded to the treatment and genotype of the animals. Normal distribution (Kolmogorov-Smirnova and Shapiro-Wilk tests) and approved homogeneity tests were analyzed. Graphs and statistical analysis were prepared using GraphPad Prism 7, InStat Software (GraphPad Software, CA, United States). Each experiment was normalized to its own control. Differences among multiple groups were assessed by one-way, two-way, and repeated-measures analysis of variance, as well as post hoc tests as stated. For independent samples, a two-tailed t test was conducted when two groups were compared. For correlation assessments, linear regression was conducted. Data are presented as mean ± SEM. Biological repetitions and details relating to all statistical analyses are presented in supplementary Tables S1–S5 below.

## Results

### A novel virally mediated small peptide delivery and expression method

**Fig. 1:**
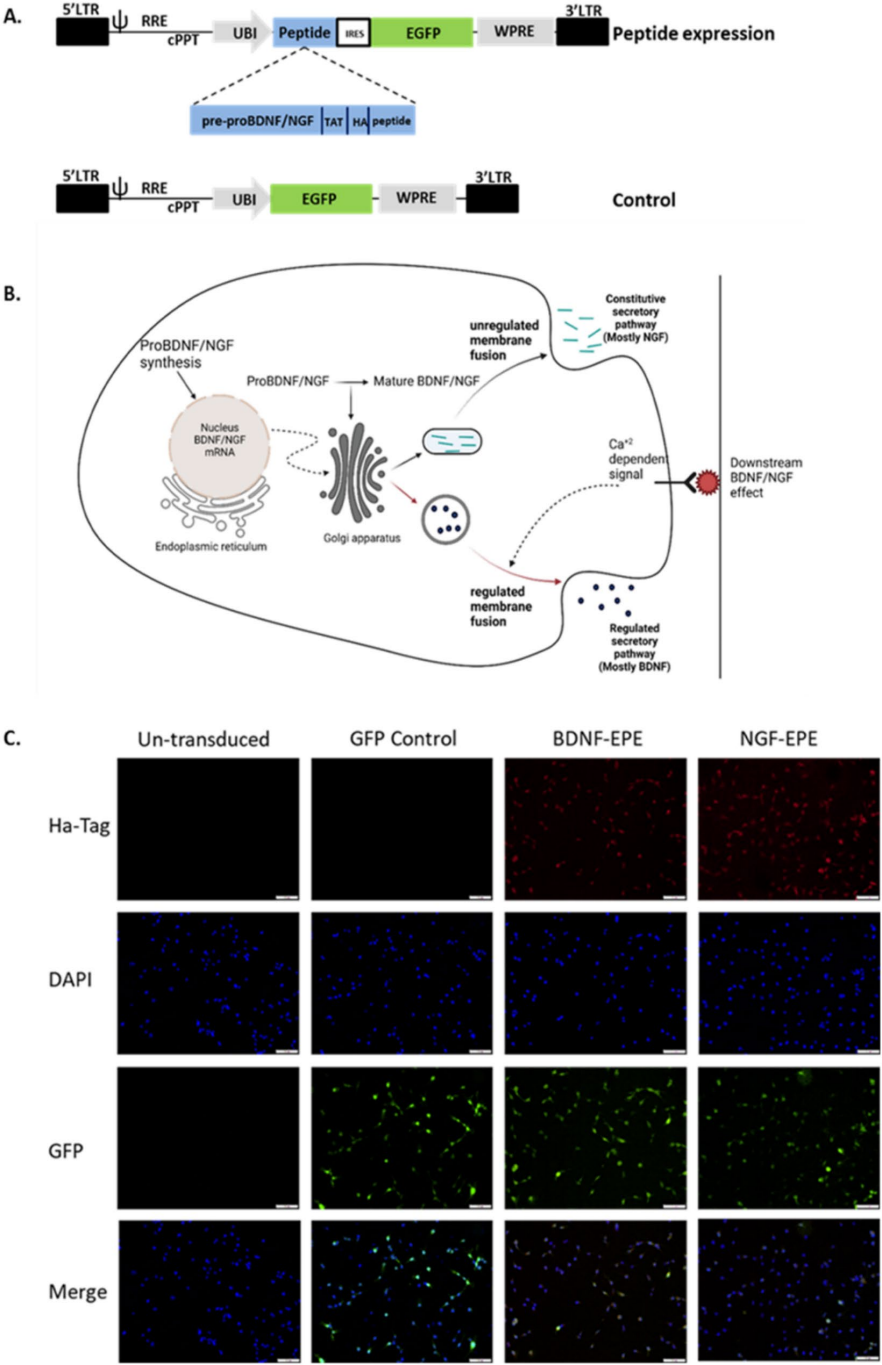
Virally mediated peptide delivery and expression system. **A**: Schematic diagram of recombinant lentiviral vectors. Control, a lentiviral vector expressing GFP; Peptide, lentiviral vector expressing peptide artificial precursor and GFP. LTR: long terminal repeat; Ψ: a packaging sequence; RRE: rev response element; cPPT: a central polypurin track sequence; IRES: internal ribosomal entry site; WPRE: a woodchuck hepatitis virus posttranscriptional regulatory element. Peptide precursor composed of the proNGF/proBDNF domain fused to TAT domain, HA tag, and peptide sequence of interest. **B**: proBDNF/NGF mRNA is translated into proBDNF/NGF protein in the endoplasmic reticulum. Pre-proBDNF/NGF is then transported into the golgi apparatus and processed to the mature form of BDNF/NGF (mBDNF/mNGF) by extracellular protein convertase 1 (PC1) within the vesicles. The secretory granules are trafficked to the sites of release in the axonal or dendritic terminals. Neurons secrete both proBDNF/NGF and mBDNF/mNGF in an activity-dependent manner (BDNF) of continuously (NGF). The tissue-type plasminogen activator (tPA) activates a plasminogen, which then cleaves the precursor molecule. Alternatively, extracellular metalloproteinases (MMP) process proBDNF/NGF to generate mBDNF/mNGF (Reviewed by Marosi & Mattson, 2014). **C**: proBDNF/proNGF-EPE or GFP LVs transduced COS7 cells were grown on PLL coated cover slips in a 12 well plate to 50% confluence. On the day of the experiment, cells were fixed with paraformaldehyde (PFA) 4%, and immunostained using anti Ha-Tag 1:200 (red), nuclei were stained by DAPI 1:5000 (blue), and viral transduction was detected by GFP fluorescence (green)

To address issues that limit the use of peptide-based drugs in vivo, and to harness the potential for targeting specific cells at distinct times, we devised a novel pharmacogenetic technique that incorporates the use of viral vectors [1]. The ongoing production of peptides within various cells, including neurons, from larger precursor molecules undergoing specific biogenesis, is well established [31]. We assumed that inducing the expression of a natural precursor through viral means would permit the artificial expression of small peptides by engaging the endogenous biogenic machinery. Therefore, we designed and cloned viral vectors that expressed the pro domains encoding sequences of BDNF/NGF precursors [32]. However, we substituted mature protein encoding regions with a sequence encoding a small peptide. We further hypothesized that in cells transduced by the virus, the transcribed transgene would undergo translation and post-translation modifications, including proteolytic cleavage, simulating the natural biosynthesis of neurotrophins. This process should lead to the endogenous production of a small, biologically active peptide of interest (US patent number: 201916971234 A; Fig. 1B). To dissociate between the multiple cytosolic and nuclear functions of ERK1/2 [33] we cloned the EPE peptide, which was shown to inhibit ERK nuclear translocation, fused to cell penetrating (TAT) and tag (HA-tag) motifs, downstream to the pre-proBDNF/proNGF sequence (Fig. 1A). We also generated a control vector expressing EGFP. To evaluate whether these vectors are functional, lentiviral (LV) particles were produced and COS7 cells, which endogenously express the mature forms of BDNF/NGF and thus harbor the appropriate biogenesis apparatus [34], were transduced with either proBDNF-EPE, proNGF-EPE or control vectors. Virally mediated expressions were analyzed by immunofluorescence allowing us to identify transduced cells through EGFP expression and to visualize the peptide precursor (HA-tag) in proBDNF/proNGF-EPE transduced cells, unlike EGFP control or un-transduced cells (Fig. 1C).

### EPE attenuates ERK nuclear translocation in COS7 cells

**Fig. 2:**
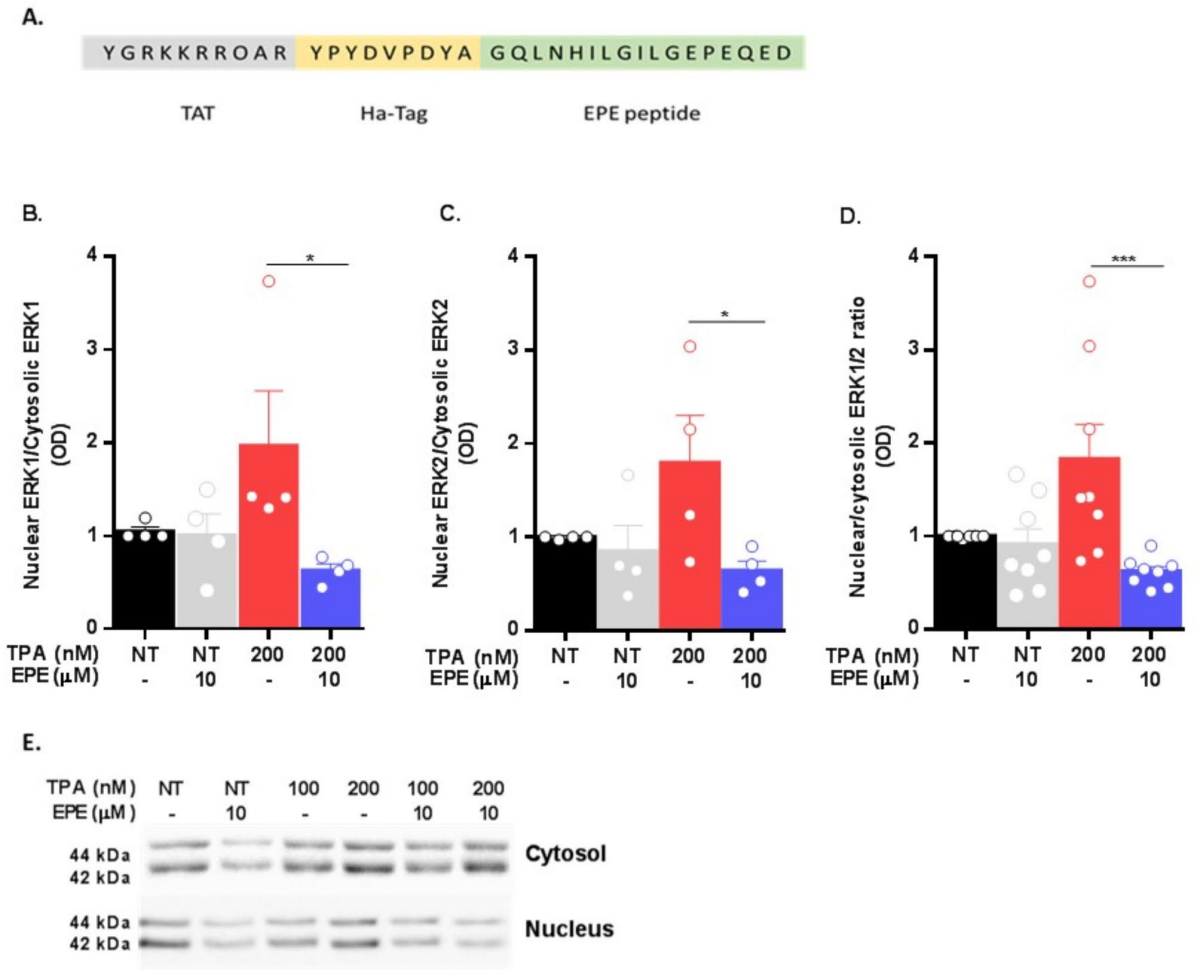
EPE peptide treatment inhibits ERK1/2 nuclear translocation in COS7 cell line. COS7 cells were grown in DMEM supplemented with 10% FBS and then serum starved (16 h, 1% FBS), pretreated either with EPE (10mM, 2 h) or left untreated as control (1% FBS). Cells were then either stimulated with 12-O-Tetradecanoylphorbol-13-acetate TPA; (200nM, 15 min) or left untreated (NT) as control. **A**: amino acid sequence of synthesized inhibitory peptide, composed of TAT as a cell penetrating motif, HA-tag and EPE sequence. **B**: Inhibition of ERK1 nuclear translocation is presented as the ratio between nuclear ERK1 levels and cytosolic ERK1 levels. Results were normalized to unstimulated cells (*N* ≥ 3, *p* = 0.0171). **C**: Inhibition of ERK2 nuclear translocation is presented as the ratio between nuclear ERK2 levels and cytosolic ERK2 levels. Results were normalized to unstimulated cells (*N* ≥ 3, *p* = 0.0157). **D**: Inhibition of the nuclear translocation of ERK1/2 is expressed as the ratio between nuclear ERK1/2 levels and cytosolic ERK1/2 levels. Results were normalized to unstimulated cells (*N* ≥ 6, *p* = 0.0006) **E**: Representative Immunoblots of COS7 cells treated with EPE (10μM, 2 h) with or without TPA stimulation (100/200nM). All data are presented as mean ± SEM

EPE treatment leads to inhibition of ERK translocation into the nucleus by disrupting its interaction with importin7 [11, 35]. We therefore aimed to validate this finding and as a first step utilized a synthetic EPE peptide, fused to TAT and HA-tag motifs, corresponding to the virally cloned peptide sequence (Fig. 2A). COS7 cells were pre-incubated with EPE (10μM, 2 h), stimulated with TPA (200nM, 15 min) and ERK1/2 levels were measured in both cytosolic and nuclear cellular fractions by western blotting (WB). In agreement with published data [10], synthesized EPE attenuates ERK1/2 transport to the nucleus as indicated by decreased nuclear/cytosolic ERK1/2 ratio (Fig. 2B; *p* = 0.0171, C; *p* = 0.0157, D; *p* = 0.0006). In addition, we evaluated ERK1/2 phosphorylation levels in whole cell lysates and as expected, treatment with EPE did not induce significant alterations (Fig. 2-1). EPE therefore specifically limits one of the various modalities associated with ERK function, its nuclear roles.

### EPE expression, facilitated by neurotrophin pro-domain processing, inhibits ERK nuclear translocation in COS7 cells

**Fig. 3:**
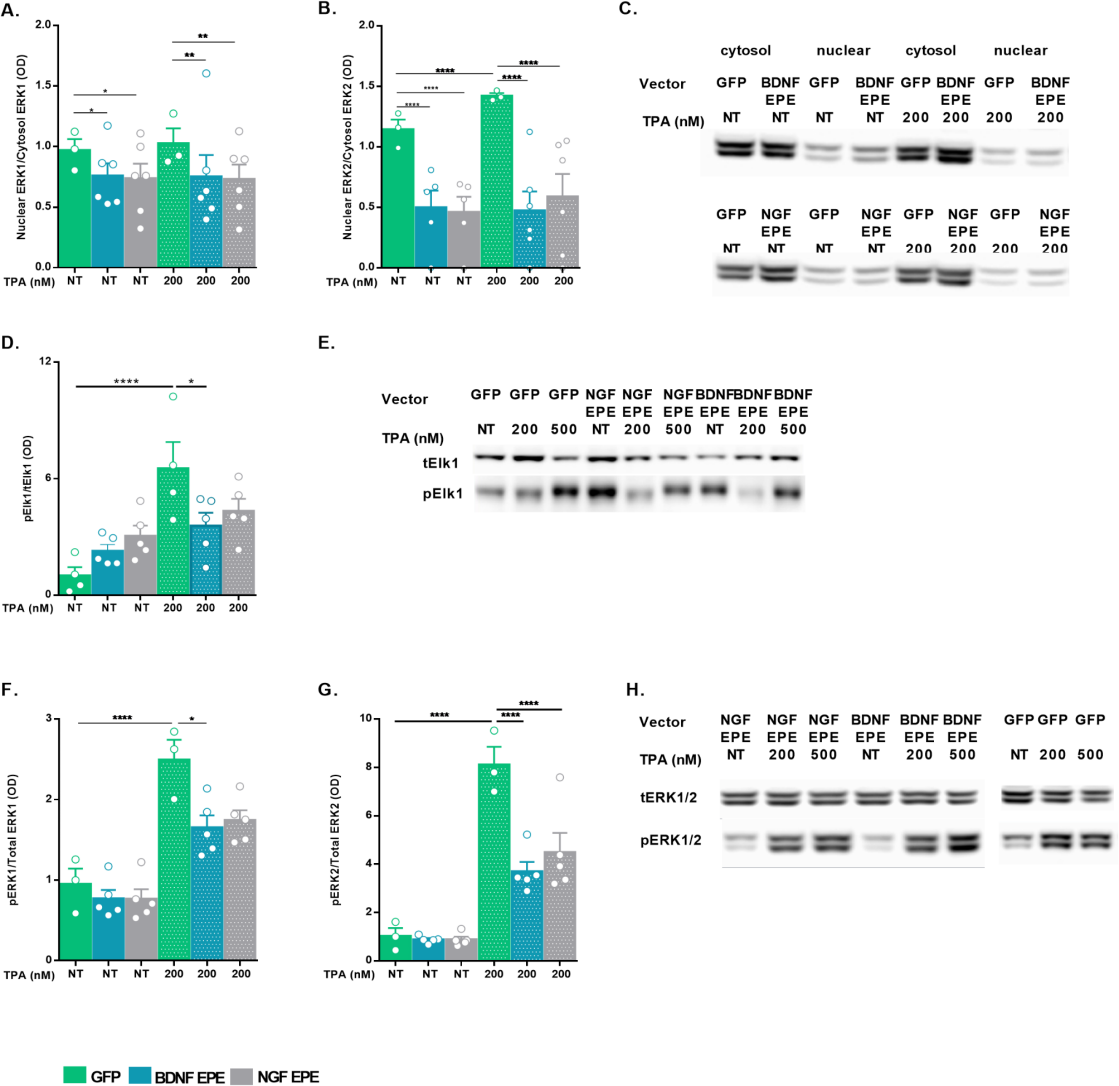
EPE expression, facilitated by neurotrophin pro-domain processing, inhibits ERK nuclear translocation in COS7 cells. COS7 cells were transduced with either proBDNF-EPE, proNGF-EPE or GFP control LVs (MOI 5) and grown in DMEM medium supplemented with 10% FBS. 24 h prior to the experiment cells were serum starved (16 h, DMEM 1%FBS) and then either stimulated with TPA (200nM, 15 min), or left untreated (NT) as control. Nuclear and cytosolic ERK1/2 protein levels were evaluated by western blot. **A**: ERK1 nuclear translocation is presented as the ratio between ERK1 nuclear levels and ERK1 cytosolic levels, results were normalized to GFP NT sample (*n* ≥ 3, NT: con versus proBDNF-EPE, *p* = 0.0177, con versus proNGF-EPE, *p* = 0.0077. TPA: con versus proBDNF-EPE, *p* = 0.0014, con versus proNGF-EPE, *p* = 0.0006). **B**: ERK2 nuclear translocation is presented as the ratio between ERK2 nuclear levels and ERK2 cytosolic levels, results were normalized to GFP NT sample (*n* ≥ 3, NT: con versus proBDNF-EPE, *p* < 0.0001, con versus proNGF-EPE, *p* < 0.0001. TPA: con versus proBDNF-EPE, *p* < 0.0001, con versus proNGF-EPE, *p* < 0.0001). **C**: Immunoblots representing the levels of ERK1/2 in the cytosol and in the nucleus **D**: Phospho-Elk1 levels are presented as the ratio between phospho-Elk1 and total Elk1. Results were normalized to GFP NT sample (*n* ≥ 4, GFP NT Versus GFP TPA 200nM: *p* < 0.0001, TPA: GFP Versus proBDNF-EPE, *p* = 0.0231). **E**: Representative immunoblots of total and phosphorylated Elk1. **F**: Phosphorylated ERK1 levels are presented as the ratio between phospho-ERK1 and total ERK1. Results were normalized to GFP NT sample (*n* ≥ 3, GFP NT Versus GFP TPA 200nM, *p* < 0.0001, TPA: GFP Versus proBDNF-EPE, *p* = 0.0208). **G**: Phosphorylated ERK2 levels are presented as the ratio between phospho-ERK2 and total ERK2. Results were normalized to GFP NT sample (*n* ≥ 3, GFP NT Versus GFP TPA 200nM, *p* < 0.0001, TPA: GFP Versus proBDNF-EPE, *p* < 0.0001, GFP Versus proNGF-EPE, *p* < 0.0001). **H**: Representative immunoblots of pERK1/2 and tERK1/2. All data are presented as mean ± SEM

Given that we replicated published data [11] and that EPE treatment triggered a well-measured biological readout (inhibition of ERK nuclear translocation), we aimed to determine whether virally mediated proBDNF/proNGF-EPE expression leads to an equivalent functional outcome. Towards this aim, COS7 cells were transduced with either proBDNF-EPE, proNGF-EPE or control LVs, stimulated with TPA (200nM; 15 min), and ERK1/2 levels were evaluated in the cytosolic and nuclear fractions using WB. The obtained results corroborated the biological activity of the virally expressed EPE peptide, as evidenced by a decreased ERK1/2 nuclear/cytosol ratio in proBDNF-EPE or proNGF-EPE transduced COS7 cells compared to GFP controls, both in TPA stimulated or unstimulated samples (Fig. 3A: NT: con versus proBDNF-EPE, *p* = 0.0177, con versus proNGF-EPE, *p* = 0.0077. TPA: con versus proBDNF-EPE, *p* = 0.0014, con versus proNGF-EPE, *p* = 0.0006, 3B: NT: con versus proBDNF-EPE, *p* < 0.0001, con versus proNGF-EPE, *p* < 0.0001. TPA: con versus proBDNF-EPE, *p* < 0.0001, con versus proNGF-EPE, *p* < 0.0001, and 3–1 A, B).

Application of small molecule-based MEK inhibitors affects the entire signaling cascade since it inhibits the kinase activity of mitogen-activated protein kinase kinase (MAPKK or MEK 1/2), the upstream activator of ERK1/2 [36]. In contrast, the use of EPE allows us to constrain selectively the nuclear roles of ERK. To confirm this assumption, we measured the activation of one of the main nuclear targets of ERK1/2, the transcription factor Elk1, which is known to be important for both regulation and initiation of proliferation and for the formation of new memories [19]. Accordingly, COS7 cells were transduced with LVs expressing the proBDNF-EPE, proNGF-EPE, or GFP control. Following transduction, cells were stimulated with TPA (200nM, 500nM; 15 min) or left untreated, and the levels of phosphorylated Elk1 were measured in cell lysates using WB. Virally mediated expression of EPE using proBDNF-EPE attenuated Elk1 phosphorylation in comparison to the GFP control (Fig. 3D-E: GFP NT Versus GFP TPA 200nM: *p* < 0.0001, TPA: GFP Versus proBDNF-EPE, *p* = 0.0231 and Fig. 3-1C, D), indicating that the nuclear functions of ERK1/2 are suppressed by EPE in COS7 cells. To confirm that ERK1/2 was activated following TPA stimulation, we analyzed its phosphorylation state in the same cell lysates. Unexpectedly, lower ERK1/2 phosphorylation levels were detected in COS7 cells transduced with proBDNF-EPE or proNGF-EPE LVs and treated with TPA compared to GFP control (Fig. 3F: GFP NT Versus GFP TPA 200nM, *p* < 0.0001, TPA: GFP Versus proBDNF-EPE, *p* = 0.0208, 3G: GFP NT Versus GFP TPA 200nM, *p* < 0.0001, TPA: GFP Versus proBDNF-EPE, *p* < 0.0001, GFP Versus proNGF-EPE, *p* < 0.0001, and Fig. 3-2A, B).

In contrast to synthetic peptide treatment (Fig. 2), lentiviral transduction leads to a stable, chronic manipulation of the target cell. Therefore, we hypothesized that prolonged lower rates of ERK1/2 in the nucleus may trigger a feedback loop, which leads to its reduced phosphorylation. MAPK phosphatases (MKPs) are key spatiotemporal regulators of MAPK signaling and are characterized by differential subcellular localization. We thus measured the levels of a cytosolic and a nuclear MKP – namely MKP-3 and MKP1, in virally transduced COS7 cells [37]. TPA stimulation resulted in significant increases in MKP3, in proBDNF-EPE, but not in proNGF-EPE expressing cells (Fig. 3-2C, *p* = 0.0145). We also found a significant correlation between ERK2 phosphorylation state and MKP3 levels (Fig. 3-2D, *p* = 0.0419), but no effect on MKP-1 levels (Fig. 3-2E).

### Impaired fear extinction in mice manipulated with proBDNF-EPE LV

**Fig. 4:**
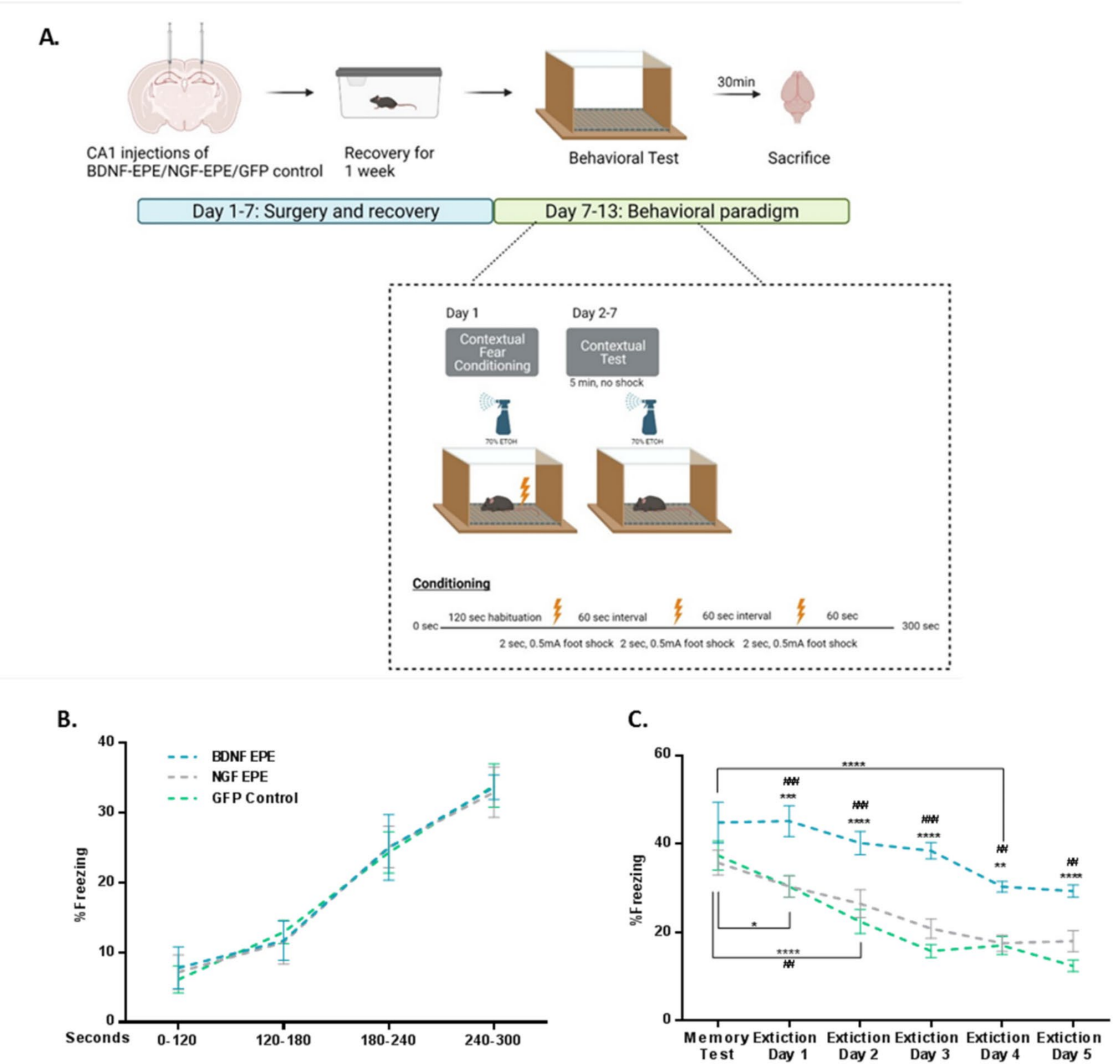
Normal fear acquisition and retrieval but impaired fear extinction in mice manipulated with proBDNF-EPE. **A**: Illustrative scheme of contextual fear conditioning displaying viral injections sites, timeline of the experiment and fear extinction protocol used. **B**: Mice manipulated with proBDNF-EPE, proNGF-EPE, or GFP control LVs exhibit comparable and normal FC learning curve. **C**: Mice injected with the proBDNF-EPE LV display higher freezing rates compared to the GFP control and proNGF-EPE experimental groups as of the third day of extinction trial. All data presented as Mean ± SEM. Mixed effects analysis (*n* ≥ 12, *Extinction Day 1*: GFP control Versus BDNF-EPE: *p* = 0.0003, NGF-EPE Versus BDNF-EPE: *p* = 0.0002. *Extinction Day 2*: GFP control Versus BDNF-EPE: *p* < 0.0001, NGF-EPE Versus BDNF-EPE: *p* = 0.0006. *Extinction Day 3*: GFP controls Versus BDNF-EPE: *p* < 0.0001 NGF-EPE Versus BDNF-EPE: *p* < 0.0001. *Extinction Day 4*: GFP control Versus BDNF-EPE: *p* = 0.0012, NGF-EPE Versus BDNF-EPE: *p* = 0.0015. *Extinction Day 5.* GFP control Versus BDNF-EPE: *p* < 0.0001, NGF-EPE Versus BDNF-EPE: *p* = 0.0056)

In light of our in vitro findings and considering the instrumental role of ERK1/2 activity in learning and memory processes [38], we explored both the applicability of our virally mediated peptide expression system in the adult mouse brain, as well as the role of nuclear ERK1/2 in learning and memory. We hypothesized that the nuclear functions of ERK1/2 are important for acquisition, retrieval, and extinction of a memory [39, 40]. To prove or refute our assumption, we used the fear conditioning (FC) learning paradigm, which relies on the CA1 region of the hippocampus for the formation of an association between a novel context and its associated auditory cue [41]. We stereotactically injected proBDNF-EPE; proNGF-EPE or EGFP LVs, into the CA1 region of the hippocampus of naïve mice (3–4 months) (see methods). Following recovery, mice underwent contextual FC, and long-term memory was evaluated 24 h after conditioning. Extinction learning was conducted over the subsequent five days. All experimental groups exhibited equivalent and normal levels of contextual fear learning acquisition (Fig. 4B). Unpredictably, considering that ERK activity is required for fear memory consolidation [42], all groups demonstrated similar high freezing rates on the memory retrieval trial (Fig. 4C). Differences between the groups emerged on the first day of extinction learning, in which proBDNF-EPE injected mice displayed significantly higher freezing rates compared to control and proNGF-EPE injected animals (Fig. 4C, *Extinction Day 1*: GFP control Versus BDNF-EPE: *p* = 0.0003, NGF-EPE Versus BDNF-EPE: *p* = 0.0002). The gap between the groups was maintained over the subsequent extinction trials (Fig. 4C: *Extinction Day 2*: GFP control Versus BDNF-EPE: *p* < 0.0001, NGF-EPE Versus BDNF-EPE: *p* = 0.0006. *Extinction Day 3*: GFP controls Versus BDNF-EPE: *p* < 0.0001 NGF-EPE Versus BDNF-EPE: *p* < 0.0001. *Extinction Day 4*: GFP control Versus BDNF-EPE: *p* = 0.0012, NGF-EPE Versus BDNF-EPE: *p* = 0.0015. *Extinction Day 5.* GFP control Versus BDNF-EPE: *p* < 0.0001, NGF-EPE Versus BDNF-EPE: *p* = 0.0056). An additional fundamental alteration was the modified slope of extinction between experimental groups. ProBDNF-EPE injected animals exhibited persistently higher freezing rates and moderate memory extinction was only observed on the fourth extinction trial, indicating impairment of extinction learning (Fig. 4C).

**Fig. 5:**
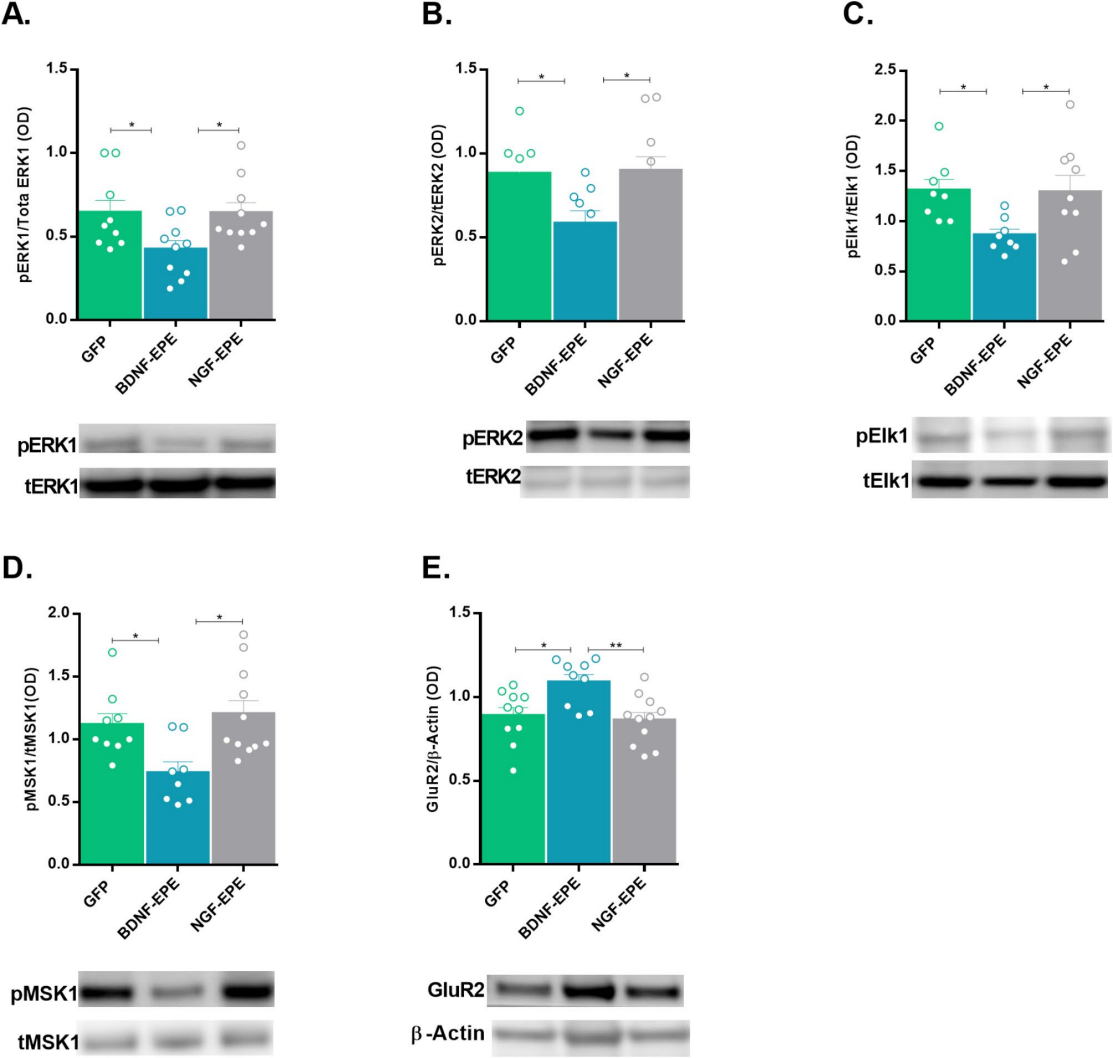
proBDNF-EPE manipulation inhibits ERK nuclear functions and induces GluR2 expression. Hippocampal CA1 regions from both hemispheres of the experimental groups were processed for western blot analysis. The upper panel represents quantified results, and representative immunoblots are displayed in the lower panel. **A**,** B**: Phospho-ERK1/2 levels are presented as the ratio between phospho-ERK1/2 and total ERK1/2. Results were normalized to control group (*n* = 8, ERK1: GFP Control versus proBDNF-EPE, *p* = 0.0010, ERK2: GFP Control versus proBDNF-EPE, *p* = 0.0273). **C**: Phospho-Elk1 levels are presented as the ratio between phospho-Elk1 and total Elk1. Results were normalized to GFP control group (*n* ≥ 8, GFP Control versus proBDNF-EPE, *p* = 0.0261). **D**: Phospho-MSK1 levels are presented as the ratio between phospho-MSK1 and total MSK1 (*n* ≥ 8, GFP Control versus proBDNF-EPE, *p* = 0.0394) **E**: GluR2 levels are presented as the ratio between GluR2 and β-Actin. Results were normalized to GFP control group (*n* ≥ 8, GFP Control versus proBDNF-EPE, *p* = 0.0131). All data are presented as mean ± SEM

To further investigate the molecular mechanisms underlying the observed behavioral effects, we isolated the CA1 subregion and processed tissue for biochemical analysis. Consistent with the in vitro results, we found a significant reduction in ERK1/2 phosphorylation (Fig. 5A, B: ERK1: GFP Control versus proBDNF-EPE, *p* = 0.0010, ERK2: GFP Control versus proBDNF-EPE, *p* = 0.0273), and of its nuclear targets Elk1 (Fig. 5C: GFP Control versus proBDNF-EPE, *p* = 0.0261) and mitogen- and stress-activated kinase 1 (MSK1; Fig. 5D: GFP Control versus proBDNF-EPE, *p* = 0.0394 ), in the proBDNF-EPE group, but not in proNGF-EPE, or control groups (Fig. 5A-D − 1). These alterations were found exclusively in mice with impaired extinction, indicating that the nuclear functions of ERK are crucial for extinction but not fear learning. In agreement with other studies [36], we further demonstrate a significant induction of GluR2 protein levels in proBDNF-EPE treated mice compared to controls (Fig. 5E: GFP Control versus proBDNF-EPE, *p* = 0.0131, Fig. 5-1F).

### Impaired CTA extinction in mice injected with proBDNF-EPE

**Fig. 6:**
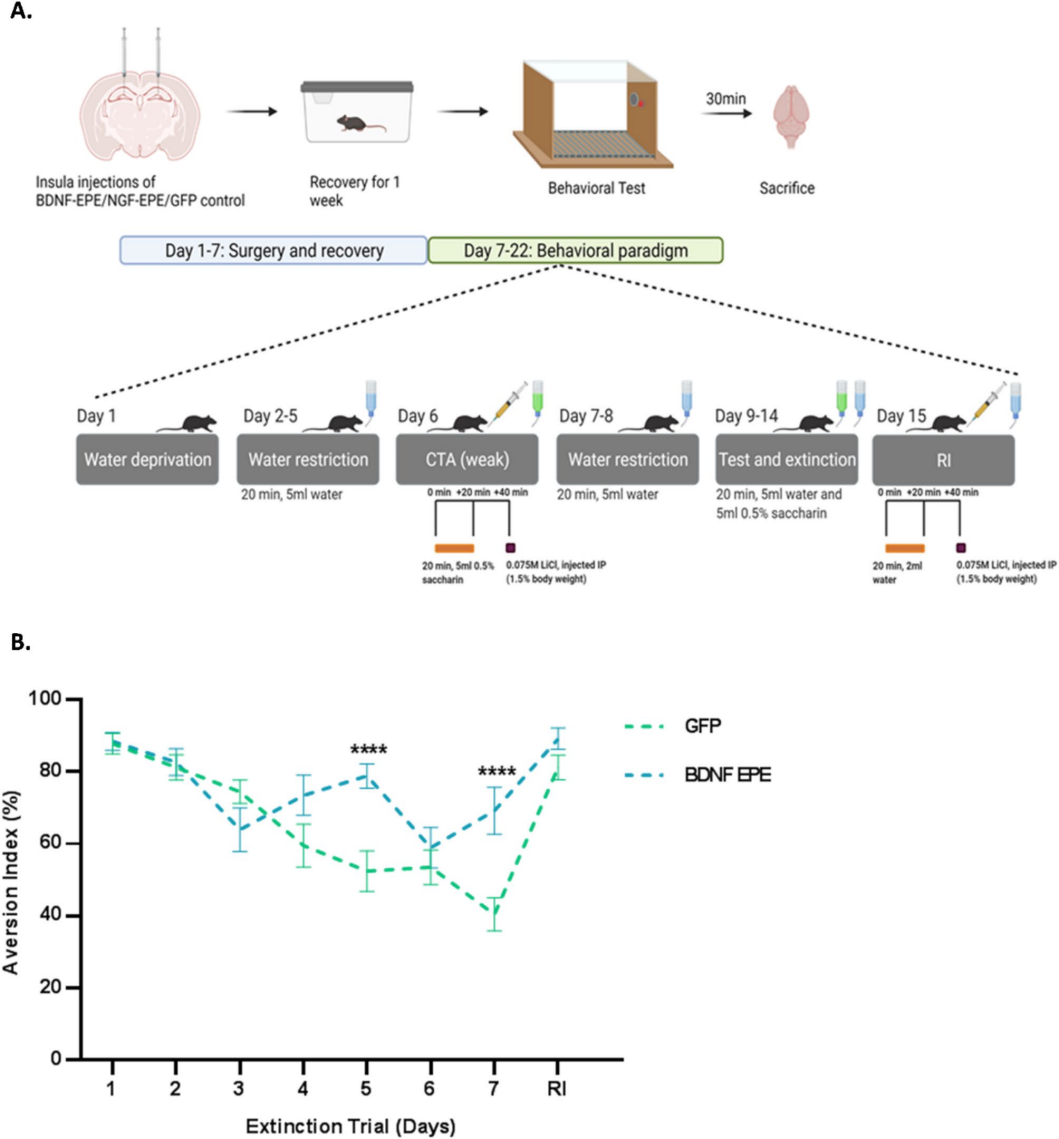
Normal CTA acquisition and impaired CTA extinction in mice manipulated with proBDNF-EPE. **A**: Representative Scheme showing injections sites, timeline of experiment and CTA protocol used for behavioral testing. **B**: Mice injected with the proBDNF-EPE LV display higher aversion index compared to the control group as of the fifth day of the extinction trial. The aversion index was defined as [ml water/ (ml water + ml saccharin) x100] consumed. Higher aversion index indicates that mice preferred water over saccharin. GFP group (*n* = 19), BDNF-EPE group (*n* = 16). Data are presented as mean ± SEM (RM Two-way ANOVA, *Extinction Day 4*: GFP control versus proBDNF-EPE: *p* = 0.0655. *Extinction Day 5: GFP* control versus BDNF-EPE: *p* < 0.0001. *Extinction Day 7*: GFP control versus BDNF-EPE: *p* < 0.0001)

Next, we asked whether extinction learning is impaired in an additional behavioral paradigm mediated by the cortex. Conditioned taste aversion (CTA) is a form of associative learning, wherein animals associate a novel taste with visceral nausea [43, 44]. Akin to fear-based memories, CTA memories are robust and long lasting, and the insular cortex (IC) subserves their acquisition and extinction [45]. Several studies showed that the application of MEK inhibitors suppressed CTA memory (reflected by lower aversion index), and that the activation of ERK in the IC is required for the acquisition of novel taste memories [20, 46]. We therefore injected proBDNF-EPE or EGFP control LVs into the IC of WT mice and following recovery, we performed a weak CTA learning followed by retrieval sessions towards extinction (Fig. 6A). In accordance with the data in fear conditioning acquisition, CTA acquisition and retrieval were not affected, whereas, both experimental groups demonstrated high and comparable aversion index (Fig. 6B). On the other hand, in the CTA extinction trials, proBDNF-EPE injected mice exhibited a significantly different slope of extinction with higher aversion index compared to the GFP control group. Differences in aversion between the proBDNF-EPE and GFP control groups reached significance between days 5 and 7 (Fig. 6B: *Extinction Day 5: GFP* control versus BDNF-EPE: *p* < 0.0001. *Extinction Day 7*: GFP control versus BDNF-EPE: *p* < 0.0001). Moreover, reinstatement of the CTA memory resulted in similar aversion between the two groups (Fig. 6B), emphasizing that the nuclear functions of ERK1/2 underlie extinction learning specifically. To confirm that the obtained behavioral results are attributed to the biological activity of the expressed peptide sequence rather than an unspecific effect of the BDNF pro-domain, we performed a CTA extinction experiment comparing mice manipulated with either LVs expressing GFP or proBDNF domain fused to a control peptide sequence. Following CTA learning, three extinction sessions were conducted in which mice were presented with saccharine and on the fourth day aversion was determined using a choice test. Both groups displayed high and comparable CTA acquisition. In addition, extinction learning was not affected as demonstrated by a significant decrease in saccharine aversion in both groups (Fig. 6-1: GFP: acquisition versus extinction, p-0.0002, proBDNF control: acquisition versus extinction, *p* = 0.0119).

### Enhanced synaptic transmission in CA1 neurons following ERK nuclear transport inhibition

**Fig. 7:**
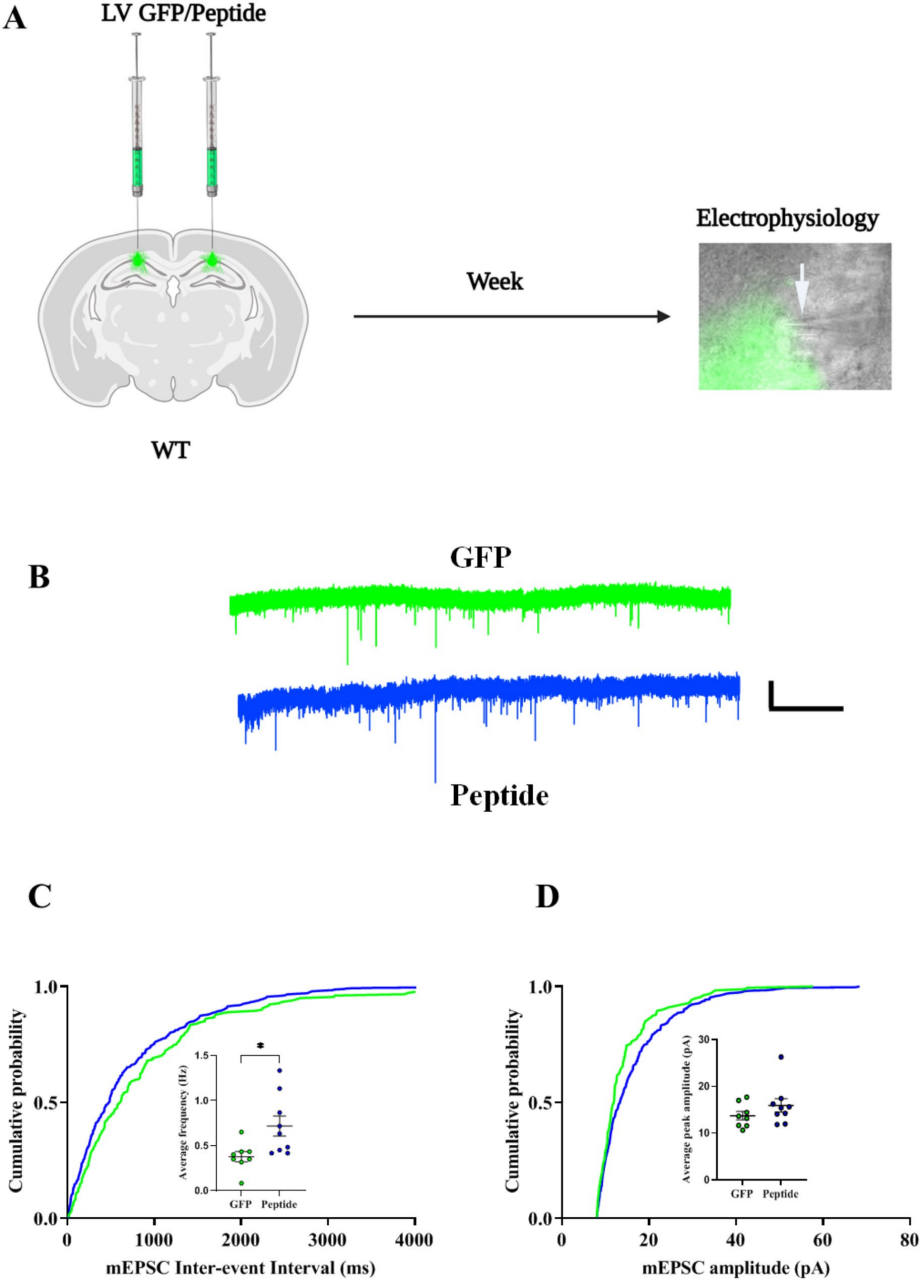
Inhibition of ERK nuclear translocation increases the frequency of mEPSCs in CA1 pyramidal neurons. **A**: The experimental design and whole-cell patch clamp recordings made from CA1 pyramidal neurons expressing GFP/proBDNF-EPE. Arrowhead showing the recording pipette. Scale 10 μm. **B**: Representative traces of mEPSCs recordings from controls (Green, an average of *n* = 8 similar traces) and peptide (blue, an average of *n* = 9 similar traces), from 4 animals each. Scale bars 20 mV and 10 s. **C**: Cumulative probability distributions and dot plots of mEPSCs inter-event intervals. Increased mEPSCs frequencies in proBDNF-EPE-treated animals (0.7167 ± 0.1111 Hz) compared to the controls (0.3771 ± 0.05554 Hz), *p* = 0.0190, Unpaired t-test. **D**: Cumulative probability distributions and dot plots of mEPSCs amplitudes. There was no significant difference in the amplitude of mEPSCs in comparing proBFND-EPE treated animals (15.92 ± 1.438 pA) and controls (13.73 ± 0.9057 pA), *p* = 0.2359, Mann Whitney test. Mann-Whitney U = 23

Our electrophysiological studies aimed to assess the impact of inhibiting ERK nuclear translocation on synaptic activity within CA1 pyramidal neurons (Fig. 7A, B). Whole-cell patch clamp recordings from CA1 pyramidal neurons expressing GFP or proBDNF-EPE revealed a significant increase in the frequency of miniature excitatory postsynaptic currents (mEPSCs) in the peptide-injected groups compared to controls (Fig. 7C, *p* = 0.0190). However, there was no significant difference in the amplitude of mEPSCs between peptide-injected groups and controls (Fig. 7D).

Collectively, our results highlight the significant impact of ERK nuclear translocation inhibition on behavioral, molecular, and synaptic levels. Our findings demonstrate that targeted disruption of ERK signaling can selectively modulate synaptic activity and memory extinction processes without affecting memory acquisition or retrieval. These insights underscore the therapeutic potential and mechanistic implications of virally delivered peptides in modulating neural functions.

## Discussion

In this study, we developed a novel approach to express targeted inhibitory peptide using viral vectors, offering a promising alternative to traditional peptide-based therapies. Our results indicate that the inhibition of ERK1/2 nuclear translocation selectively impairs memory extinction while sparing acquisition and retrieval. This finding builds on previous research that identified ERK1/2 signaling as a crucial modulator of learning and memory [8, 38]. However, unlike prior studies using small-molecule inhibitors, our approach offers a more localized and chronic inhibition of nuclear ERK1/2 signaling, enabling more precise modulation of ERK1/2’s multifunctional roles.

In the realm of therapeutics, peptides are increasingly viewed as a promising category of drugs. Defined as short chains of 2 to 50 amino acids, peptides offer a compelling blend of characteristics found in both small molecules, as well as larger biomolecules, such as antibodies. Their versatile nature lends to their ability to modulate challenging targets, such as protein-protein interactions and extensive protein surfaces without clear binding pockets. This is primarily due to their high selectivity, specificity, and strong binding affinity, which reduces the likelihood of undesirable side-effects and off-target effects. Despite the traditionally perception of their pharmacokinetic limitations, peptides are proving to be an effective approach for drug development, as evidenced by the fact that over 80 peptide drugs have already been approved for various therapeutic indications. However, challenges persist in the development of peptide therapeutics, particularly concerning their bioavailability and membrane permeability. To facilitate their broad acceptance in the therapeutic field, ongoing research seeks innovative strategies to overcome these hurdles [47].

To overcome the inherent challenges of peptide-based inhibitors [1, 2] and to harness the advantages of viral vectors, we developed a novel, virally mediated approach to deliver and express a peptide of interest. Viral vectors exhibit tropism, allowing for targeted expression in specific cell types using promoters, and offer temporal precision due to their reliable and reproducible infection profiles [23]. We exploited the cellular features of neurotrophin biosynthesis machinery, in which naturally produced peptides are processed and protected from biodegradation, to express the EPE peptide, which specifically inhibits ERK1/2 translocation from the cytosol to the nucleus [11, 12]. Surprisingly, we found that inhibition of the nuclear functions of ERK1/2 does not affect learning or memory retrieval; but is in fact crucial for the extinction of aversive memories. One potential consideration is the absence of an unconjugated EPE control. However, previous studies have shown that unconjugated EPE lacks stability to effectively inhibit ERK nuclear translocation [10]. Moreover, we verified that both unconjugated and virally expressed EPE lead to equivalent biochemical outcomes in vitro (i.e., inhibition of ERK1/2 nuclear translocation. Our viral vector approach ensured sustained intracellular expression of EPE, accounting for any effects related to pro-domain expression while isolating the impact of inhibition of ERK nuclear translocation. Importantly, our study demonstrated the feasibility of using viral vectors harboring a peptide expression cassette, showcasing the approach’s versatility and broad applicability for diverse peptide therapeutics. Notably, while a direct LV-EPE comparison was not performed in this study, we assume that the expressed peptide precursor undergoes cleavage mimicking the endogenous biosynthesis of neurotrophins. In addition, our molecular analyses confirm robust inhibition of nuclear ERK activity, supporting the effectiveness of this delivery system. Future studies could further quantify the relative efficiencies of these approaches to refine peptide-based inhibitory strategies for targeted molecular interventions.

Peptide-based drug delivery systems have primarily been applied as peptide-drug conjugates and injectable biodegradable particles. However, controlled drug delivery systems that can effectively deliver peptide-based drugs without significant side effects are required [48]. Here, we demonstrate the potential use of LVs as a peptide-delivery system, which can be easily adapted to any viral vector apparatus. At present, approaches based on viral vectors utilize either adenoviruses, adeno-associated viruses, or LVs, and have directed the way in preclinical and clinical achievements in the past two decades. In recent years, drugs based on viral vectors in a variety of designs and purposes have gained regulatory approval [49]. Still, challenges related to biosafety limit these approaches. Advancements in the safety and efficiency of viral vector-based gene delivery systems are making gene therapy increasingly viable, however fully addressing these biosafety concerns would be imperative.

ERK is a highly promiscuous kinase that phosphorylates many substrates [50]; and its distinct roles in learning processes remain under intense investigation. As shown in previous studies and corroborated here, synthetic EPE inhibited ERK nuclear transport as evidenced by decreased nuclear/cytosolic ERK ratio as well as blunted Elk1 phosphorylation in response to stimulation [8]. LV-mediated transduction of proBDNF/NGF-EPE transduction in COS7 cells also resulted in suppressed ERK nuclear localization under both basal and stimulated conditions. However, unlike synthetic EPE treatment, our approach resulted in significant decreases in phosphorylated ERK in transduced cells (Fig. 3F, G and H and S3-2A, S3-2B). This unexpected result may have been the consequence of differences in treatment duration and experimental approaches. Application of synthetic EPE was done two hours prior to cell lysis, while viral transduction experiments persisted for at least a week, resembling a chronic treatment, which would induce the engagement of compensatory feedback loops (i.e., activation of phosphatases). Indeed, we found that cytosolic MAPK Phosphatase-3 (MKP-3) expression was increased in transduced cells. MKP-3 is a dual-specificity phosphatase [51] that acts as a negative regulator of the MAPK pathway, which modulates the duration and magnitude of signaling responses [52, 53]. Our results may indicate that in rendering phosphorylated ERK unable to enter the nucleus, LV proBDNF/proNGF-EPE transduction led to disproportionate increases in MKP-3, suppressing ERK phosphorylation in both the nucleus and cytosol. Importantly, while our approach relies on the proteolytic processing of neurotrophin pro-domains for peptide expression and stability, our data does not support a direct relationship between BDNF/NGF biogenesis and EPE production. To clarify, EPE production in this system is dictated by viral vector-mediated expression and subsequent pro-domain processing, rather than being regulated by endogenous BDNF or NGF expression levels or signaling pathways. Interestingly, LV-mediated EPE transduction paradoxically increased Elk1 phosphorylation under baseline conditions, which was unaffected by TPA stimulation. This latter finding would further suggest that alternative pathways may partially compensate for ERK-dependent Elk1 regulation under resting conditions [54], while activity-dependent transcriptional modulation remains significantly impaired. These findings underscore the complex interplay between ERK signaling, compensatory feedback mechanisms, and transcriptional regulation in COS7 cells.

LV proBDNF-EPE, but not LV proNGF-EPE, delivery at the CA1 disrupted fear memory extinction, which correlated with the suppression of ERK phosphorylation in this region. Our intervention did not affect the acquisition, retrieval, or reinstatement of fear memories, implicating the reduction in nuclear ERK interfered with specific, brain-region-dependent molecular mechanisms that are fundamental to extinction. Consistent with our findings in COS7 cells, Elk1 phosphorylation at the CA1 region was suppressed in response to LV proBDNF-EPE manipulation, alongside the suppression of MSK1 phosphorylation (Fig. 5). MSK1 is directly involved in the regulation of gene expression since it phosphorylates, stabilizes, and activates various transcription factors recruited during contextual fear conditioning [55]. However, evidence indicates that while MSK1 participates in BDNF signaling by phosphorylating CREB under basal conditions, it is not required for hippocampal spatial memory formation [56, 57]. Considering that memory extinction increases BDNF biogenesis in relevant brain structures [58, 59], it is possible that enhanced engagement of this cellular machinery in the insula or hippocampus in proBDNF-EPE manipulated animals, led to enhanced EPE production as a function of extinction learning, contributing to the observed differences.

Our findings further indicate that disrupting the ability of neurons to encode experience through changes in gene expression, affects their capacity to modify previously acquired information. Importantly, the observed deficits in extinction learning cannot be attributed to neurotrophic factor overexpression, as our constructs did not produce mature BDNF or NGF, and our control demonstrated that pro-BDNF domain expression alone did not influence behavior. Previous studies have indicated that Elk-1 phosphorylation by ERK is crucial in the ability of the brain to link experience-driven activity and plasticity with gene expression and regulation [13, 60]. During memory acquisition, Elk-1 activation supports the formation of new synaptic connections by initiating the transcription of genes required for long-term potentiation (LTP) in both hippocampal and cortical regions [61, 62]. This process is essential for the consolidation of new memories through the strengthening of synaptic connections. In contrast, during memory extinction, Elk-1 phosphorylation aids in the remodeling and weakening of these synaptic connections, facilitating the consolidation of new memories [54, 63, 64]. As our intervention blunted memory extinction, but not its acquisition and retrieval, it is tempting to speculate that Elk-1 phosphorylation via ERK constitutes a molecular mechanism that enables the reshaping of experience through expression-driven changes in gene expression.

Fear memory extinction can be hindered by changes in the expression and post-translational modification of ion channels and receptor systems that contribute to synaptic plasticity [65]. GluR2-containing α-amino-3-hydroxy-5-methyl-4-isoxazolepropionic acid (AMPA) receptors regulate synaptic strength, and their upregulation stabilizes synapses, maintaining fear responses [66]. Notably, pharmacological inhibition of the MEK/ERK pathway has been previously shown to be associated with increased GluR2 expression, suggesting that ERK signaling directly influences GluR2 levels [36]. The persistence of freezing behavior observed in LV proBDNF-EPE-manipulated mice undergoing extinction tended to correlate (*p* = 0.059) with increased expression of GluR2 in CA1 region of the hippocampus. This finding aligns with previous studies where GluR2 expression at the hippocampus was shown to increase following fear conditioning, and to decrease in response to extinction training [67]. Migues et al. (2016) suggested that synaptic removal of GluA2/AMPARs supports the natural forgetting process, emphasizing the role of GluR2 in memory persistence and extinction. Similarly, more recent work investigating GluR2 endocytosis in the amygdala, would indicate a shared critical mechanism for the reconsolidation, updating and extinction of fear memories [68]. The ability of our peptide to modulate specific aspects of plasticity-related signaling cascades provides a powerful tool for investigating the molecular underpinnings of memory persistence. Our data using this virally expressed, neurotrophin-processing-based EPE peptide highlights the potential for targeted modulation of these pathways to influence fear memory extinction. The correlation between increased GluR2 expression and disrupted extinction in response to peptide-driven inhibition of ERK’s nuclear functions suggests the existence of a potential therapeutic axis for the extinction of traumatic memories. Having said that, the upregulation of phosphatases like MKP-3 in response to chronic ERK inhibition highlights the need to consider feedback mechanisms in therapeutic designs. Integrating our approach with inducible systems could offer a more controlled and precise method to study and modulate these pathways, potentially mitigating compensatory mechanisms and enhancing therapeutic efficacy [69].

Importantly, our approach revealed that disruption of ERK nuclear functions, consistent with its role in plasticity- and activity-dependent transcriptional regulation, led to changes in synaptic function [20, 38]. This aligns with studies demonstrating that the ERK-dependent phosphorylation of Elk-1 is a crucial component for transcriptional control tied to synaptic plasticity [54, 70]. The increased mEPSC frequency observed in the CA1 of proBDNF-EPE treated animals suggests changes in presynaptic release probability and overall synaptic activity (Fig. 7). Reduced Elk-1 and MSK1 phosphorylation in treated trained animals, supports the idea that disrupting ERK’s nuclear activity biases synaptic states towards stability rather than adaptability, potentially contributing to cognitive rigidity. This is further consistent with our finding that proBDNF-EPE-treated animals were capable of learning and memory retrieval in both CTA and FC paradigms but exhibited significant resistance to aversive memory extinction. By altering ERK’s transcriptional regulation of plasticity-related genes, such as those involving GluR2, the treatment may favor persistent synaptic strength at the cost of adaptability [71]. GluR2-containing AMPA receptors play a crucial role in regulating synaptic strength and plasticity, and their upregulation, could drive increases in synaptic activity [66]. Additionally, increased MKP-3 expression in response to EPE treatment might modulate synaptic transmission and cognitive flexibility [72, 73]. MKP-3, by dephosphorylating ERK, can shift signaling balances and enhance synaptic activity as a compensatory mechanism [52]. These findings underscore the relationship between Elk-1 and genes necessary for synaptic remodeling [13, 60, 74], and highlight the regulation of GluR2 expression [75]. Given the critical role of AMPA receptors in excitatory synaptic transmission and their tight regulation ERK nuclear signaling disruptions could significantly impact synaptic function and cognitive health [76, 77].

Our findings highlight a crucial link between the nuclear functions of ERK1/2 and the regulation of synaptic plasticity through the modulation of AMPA receptors. The observed increase in GluR2 expression in the CA1 region following EPE manipulation suggests that nuclear ERK1/2 plays a significant role in maintaining synaptic strength and remodeling connections during memory extinction. The correlation between disrupted extinction and elevated GluR2 levels aligns with previous studies, which indicate that ERK1/2-mediated transcriptional regulation is essential for dynamic synaptic adjustments. Given the central role of AMPA receptors in excitatory synaptic transmission, these results imply that targeted inhibition of ERK1/2’s nuclear functions could selectively impact synaptic remodeling without compromising overall memory consolidation. This precise modulation of synaptic plasticity presents new opportunities for exploring treatments for neuropsychiatric conditions characterized by altered synaptic dynamics and persistent memories, such as post-traumatic stress disorder or addiction. Recent studies provide further insight into the relevance of peptide-based modulation in complex signaling pathways. Research on SAIF, a shark-derived peptide, demonstrated its ability to inhibit the VEGF-VEGFR2-ERK signaling pathway, effectively reducing tumor angiogenesis in vitro and in vivo [78]. Additionally, in silico studies in investigating the potential utility of MAPK1 inhibition for Alzheimer’s treatment through computational modeling and virtual screening, identified potential peptide epitopes capable of disrupting pathological MAPK signaling that contributes neurodegenerative disease mechanisms [79]. This work underscores the broader applicability of peptides in selectively modulating specific signaling modalities.

Despite these promising findings, the study also underscores the complexity of targeting multifunctional signaling pathways like ERK1/2. The activation of compensatory mechanisms, such as MKP-3 upregulation in response to chronic ERK inhibition, suggests the potential for feedback loops that could diminish the long-term efficacy of interventions. To address these issues, integrating inducible viral systems could allow for finer temporal regulation of peptide expression, minimizing compensatory responses and optimizing therapeutic outcomes. Furthermore, applying this approach to different brain regions and employing promoter-specific vectors would enable a more detailed understanding of the cell-type-specific roles of nuclear ERK1/2 in neuroplasticity and cognitive flexibility. Such refinements could broaden the therapeutic applicability of this strategy, facilitating its translational potential towards signaling modalities of interest.

## Electronic supplementary material

Below is the link to the electronic supplementary material.

Supplementary Material 1

## Data Availability

Data is provided within the manuscript or supplementary information files.

## Abbreviations

ACSF: Artificial Cerebrospinal Fluid
AMPA: α-amino-3-hydroxy-5-methyl-4-isoxazolepropionic Acid
BDNF: Brain-Derived Neurotrophic Factor
CA1: Cornu Ammonis 1 (region of the hippocampus)
CCD: Charge-Coupled Device
COS7: Monkey Kidney Fibroblast Cell Line
CS: Conditioned Stimuli
CTA: Conditioned Taste Aversion
DAPI: 4’,6-Diamidino-2-Phenylindole
DDW: Double Distilled Water
DMSO: Dimethylsulphoxide
EGFP: Enhanced Green Fluorescent Protein
ERK: Extracellular Signal-Regulated Kinase
FC: Fear Conditioning
GFP: Green Fluorescent Protein
GluR2: Glutamate Receptor 2
HA-tag: Hemagglutinin Tag
IC: Insular Cortex
LTP: Long-Term Potentiation
LV: Lentiviral Vector
MAPK: Mitogen-Activated Protein Kinase
MEK: Mitogen-Activated Protein Kinase Kinase
mEPSC: Miniature Excitatory Postsynaptic Current
MKP: Mitogen-Activated Protein Kinase Phosphatase
MSK1: Mitogen- and Stress-Activated Kinase 1
NTS: Nuclear Translocation Signal
pA: Picoampere
PBS: Phosphate Buffered Saline
RT: Room Temperature
SEM: Standard Error of the Mean
TBS-T: Tris-Buffered Saline with Tween 20
TF: Transcription Factor
TPA: 12-O-Tetradecanoylphorbol-13-acetate
TTX: Tetrodotoxin
WB: Western Blotting
WT: Wild Type
