# Supplementary material for: Virally mediated expression of a biologically active peptide to restrain the nuclear functions of ERK1/2 attenuates learning extinction but not acquisition
Source: Mol Brain. 2025 Mar 14;18:19. doi: 10.1186/s13041-025-01190-1 (PMC11908084; doi:10.1186/s13041-025-01190-1)
Supplement: Supplementary file 1 — Supplementary Material 1 [file 13041_2025_1190_MOESM1_ESM.pdf]

Supplementary material

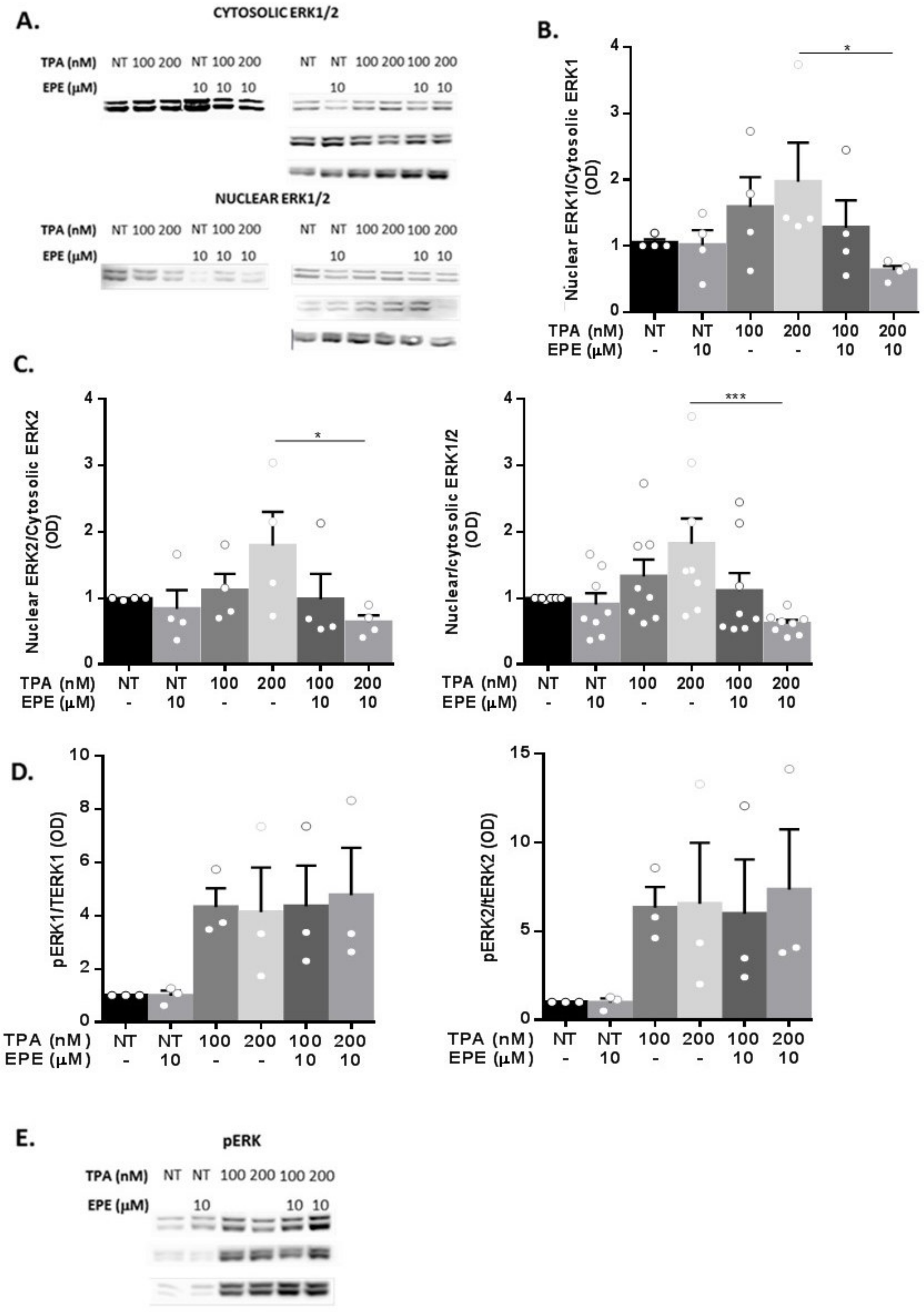

Figure 2-1. EPE peptide inhibits ERK1/2 nuclear translocation in COS7 cell line. COS7 cells were pre-treated with EPE (10μM, 2hr) followed by TPA stimulation (100 or 200nM, 15min). Cell lysis and fractionation were done and levels of ERK1/2 in the nucleus and cytosol were evaluated using WB. **A:** Full length uncropped original immunoblots of nuclear and cytosolic ERK1/2 are presented from four different experiments. **B, C**: Nuclear translocation of ERK1/2 is presented as the ratio between nuclear and cytosolic ERK1/2 levels. (Ordinary one-way ANOVA, n=4, ERK1: TPA, 200μM versus TPA, 200μM+EPE p=0.0171; ERK2: TPA, 200μM versus TPA, 200μM+EPE p=0.0157). **D:** The phosphorylation levels of ERK1/2 were analyzed in COS7 cell lysates and presented as pERK levels normalized to tERK levels. Means ± SEM are shown (Ordinary one-way

ANOVA, n=3). (E) Full length uncropped original immunoblots of pERK are shown from 3 different experiments.

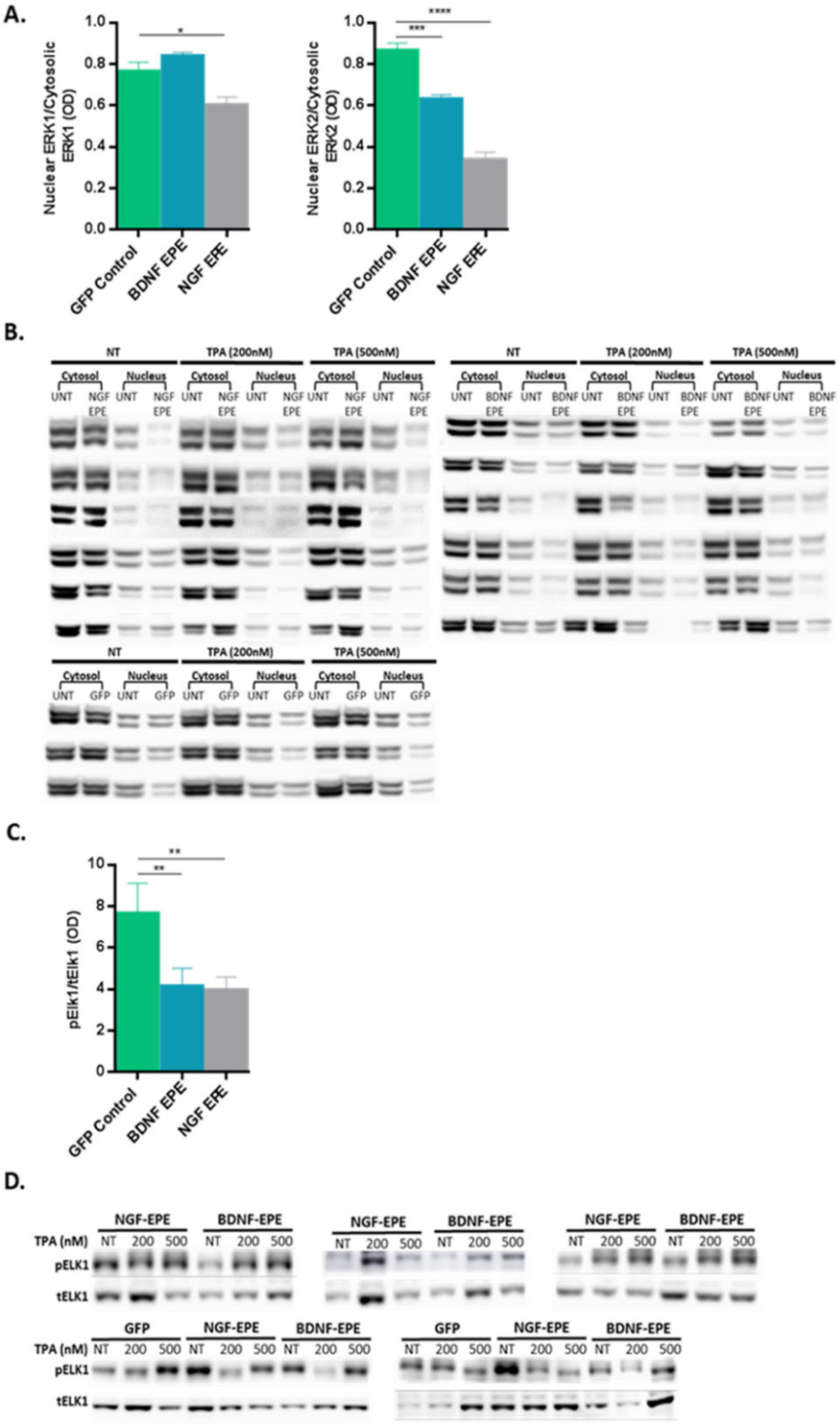

Figure 3-1: EPE expression, facilitated by neurotrophin pro-domain processing, inhibits ERK nuclear translocation in COS7 cell line. COS7 cells were transduced with either proBDNF-EPE, proNGF-EPE or GFP

control LVs (MOI 5) and grown in DMEM medium supplemented with 10% FBS. 24hr prior the experiment cells were serum starved (16h, DMEM 1%FBS) and then either stimulated with TPA (500nM, 15min), or left untreated (NT) as control. **A:** Nuclear and cytosolic ERK1/2 protein levels were evaluated by western blot and presented as the ratio between nuclear and cytosolic ERK1/2 levels. Means ± SEM are shown (Ordinary one-way ANOVA, n≥3, ERK1: GFP versus BDNF-EPE p=0.0256; ERK2: GFP versus BDNF-EPE p=0.0009, GFP versus NGF-EPE p<0.0001). **B**: Full length uncropped original immunoblots of nuclear and cytosolic ERK1/2 are shown from 5 different experiments for proBDNF/NGF-EPE LVs transduction and 3 different experiments for GFP control. **C**: Activation of Elk1 was analyzed in COS7 cells transduced with either proBDNF/NGF-EPE or GFP control LVs and presented as pElk1 normalized to total Elk1 levels. Mean ± SEM are shown (Ordinary one-way ANOVA, n≥3, GFP versus BDNF-EPE p=0.0036, GFP versus NGF-EPE p=0.0022). **D:** Full length uncropped original immunoblots of total Elk1 and pElk1 are presented.

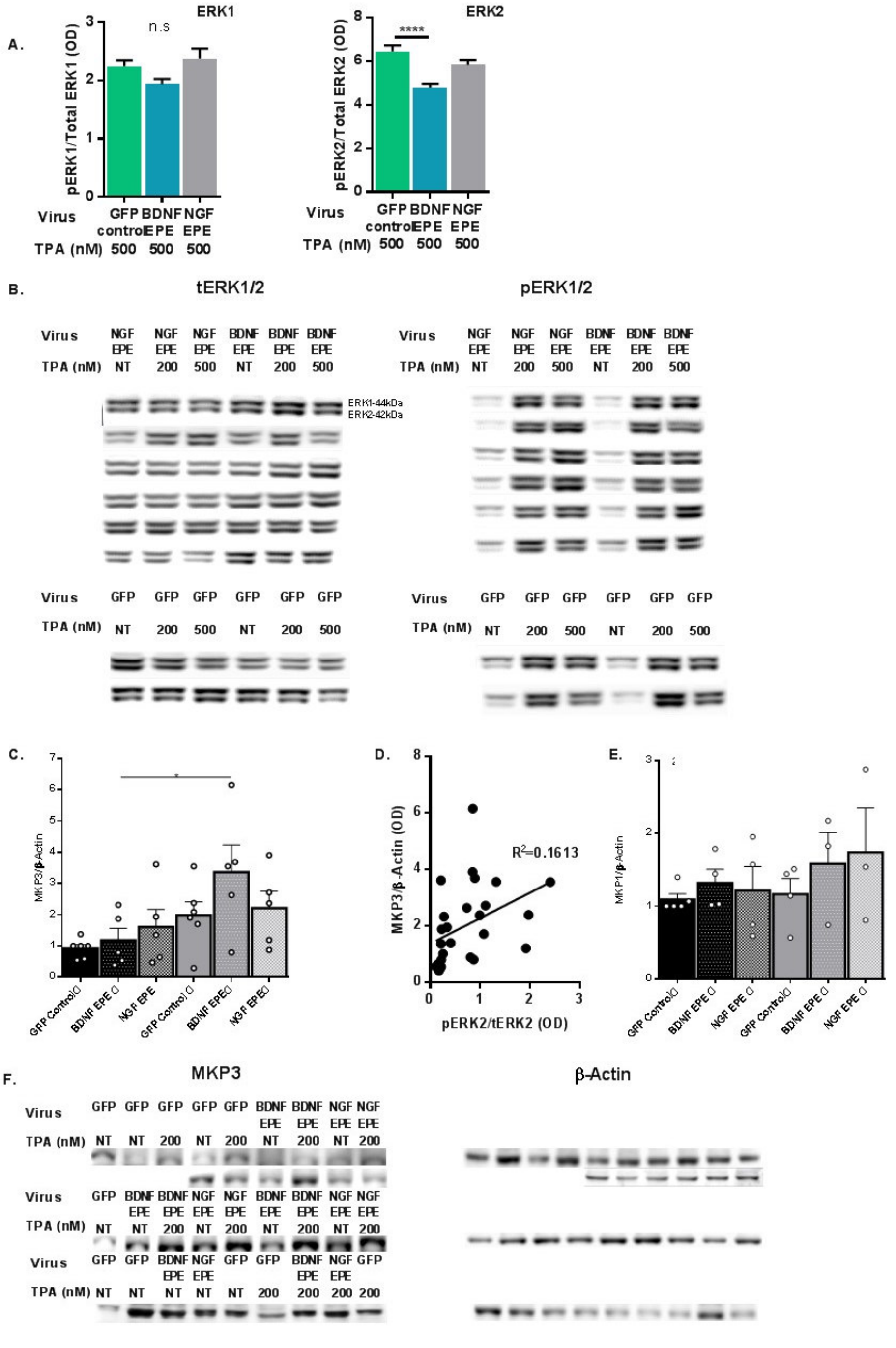

Figure 3-2: EPE expression, facilitated by neurotrophin pro-domain processing, results in reduced ERK2 phosphorylation in COS7 cells. A: COS7 cells were transduced with either proBDNF-EPE, proNGF-EPE or GFP control LVs (MOI 5) and grown in DMEM medium supplemented with 10% FBS. 24hr prior the experiment cells were serum starved (16h, DMEM 1%FBS) and then either stimulated with TPA (200nM or 500nM, 15min), or left untreated (NT) as control. A. ERK1/2 phosphorylation levels are presented as the ratio between pERK1/2 to tERK1/2. Means ± SEM are shown (Ordinary one-way ANOVA, n≥3, $p<0.0001$). **B:** Full length uncropped original immunoblots of phosphorylated and total ERK1/2 are shown from five different experiments for proBDNF/NGF-EPE and two different experiments for GFP control. **C:** MKP-3 levels are presented as the ratio between MKP-3 and β-Actin. Means ± SEM are shown (Ordinary one-way ANOVA, n=3, $p=0.0145$). **D:** Correlation analysis between MKP-3 and pERK2 levels. (Linear regression of correlation, p=

0.0419). **E:** MKP1 levels are presented as the ratio between MKP1 and β-Actin. Means ± SEM are shown (Ordinary one-way ANOVA, n=3). **F:** Full length uncropped original immunoblots of MKP-3, and β-Actin are shown from three different experiments.

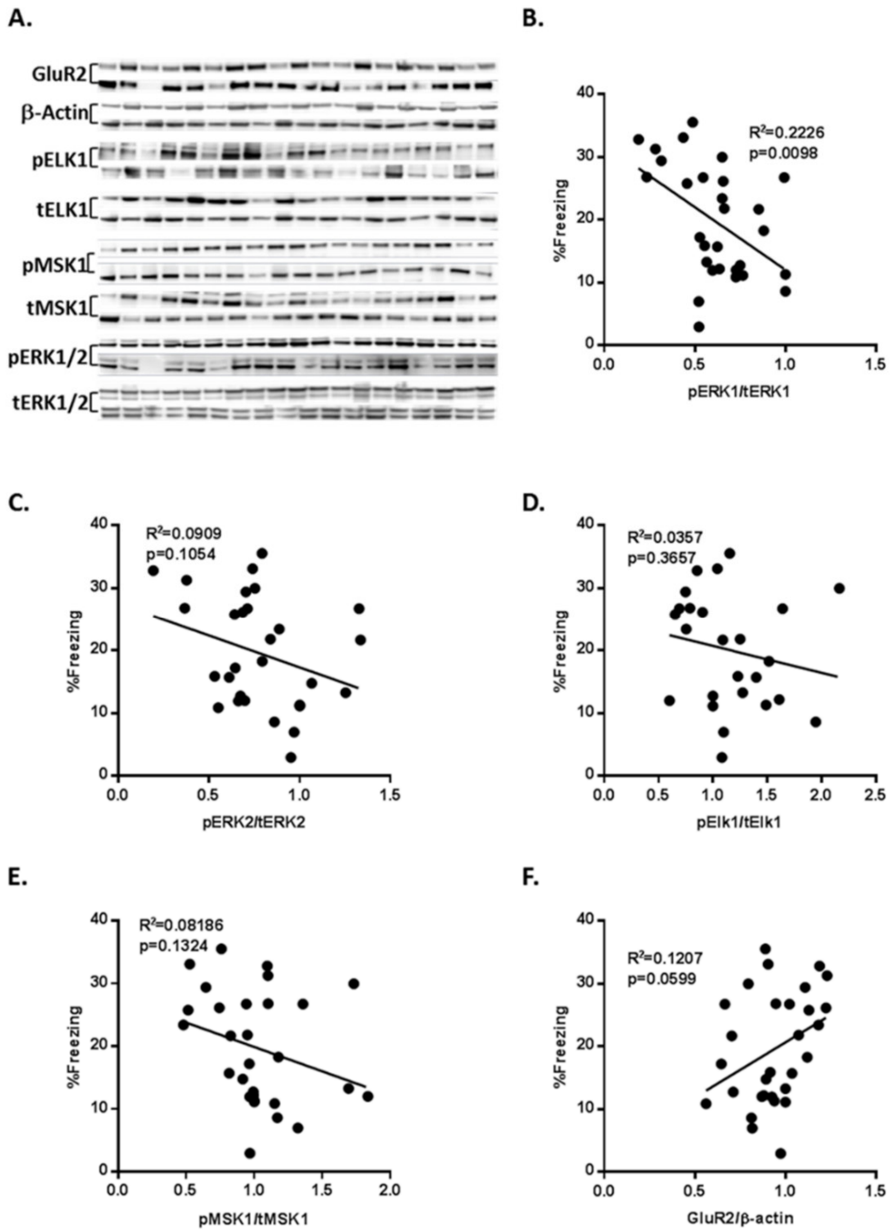

Figure 5-1: ProBDNF-EPE manipulation inhibits ERK nuclear functions and induces GluR2 expression. Hippocampal CA1 regions from both hemispheres of the experimental groups (proBDNF-EPE, proNGF-EPE, and GFP control) were processed for western blot analysis. **A:** Full length uncropped original immunoblots of the analyzed proteins are presented. **B-F:** Correlation analysis between freezing percentage and the indicated proteins (Linear regression of correlation, n≥8).

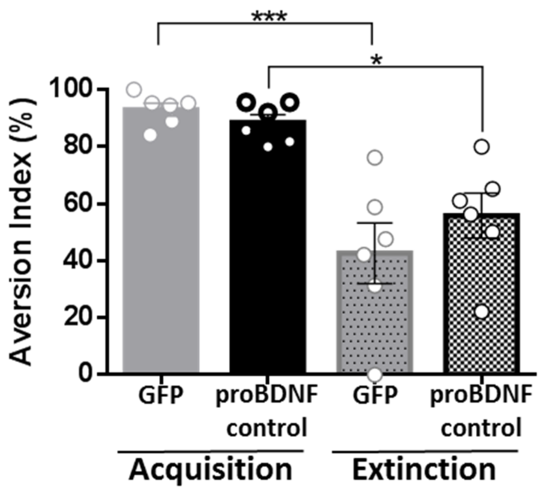

Figure 6-1: Control peptide expression, facilitated by neurotrophin pro-domain processing, results in normal CTA acquisition and extinction. Mice injected with the proBDNF-control peptide and GFP LVs display comparable aversion index in both acquisition and extinction trials. The aversion index was defined as [ml water/ (ml water + ml saccharin) x100] consumed. GFP group (n=6), proBDNF-control group (n=6). Data are presented as mean ± SEM (One-way ANOVA; GFP: acquisition versus extinction, p=0.0002, proBDNF control: acquisition versus extinction, p= 0.0119).

Table S1. Detailed statistical analysis, related to Figure 2

**Figure 2**	**Biological repetitions**	**Statistics**	**Post hoc test (**Sidak's multiple comparisons**)**
2B	4	One-way ANOVA: $F_{(5, 18)} = 1.716$, p=0.1820.	TPA 200nM Versus TPA 200 nM +10µM EPE: $T_{18} = 2.626$, p=0.0171.
2C	4	One-way ANOVA: $F_{(5, 18)} = 1.655$, p=0.1965.	Sidak's multiple comparisons test: TPA 200nM Versus TPA 200 nM +10µM EPE: $T_{18} = 2.668$, p=0.0157.
2D	8	One-way ANOVA: $F_{(5, 42)} = 3.214$, p=0.0152.	Sidak's multiple comparisons test: TPA 200nM Versus TPA 200 nM +10µM EPE: $T_{42} = 3.705$, p=0.0006.

Table S2. Detailed statistical analysis, related to Figure 3

**Figure 3**	**Biological repetitions**	**Statistics**	**Post hoc test (**Sidak's multiple comparisons**)**
3A	GFP: 3<br>BDNF-EPE: 6<br>NGF- EPE: 6	One-way ANOVA:<br>$F_{(8, 36)} = 6.871$, p<0.0001.	**GFP TPA 200nM Versus BDNF-EPE TPA 200 nM**: T_{36}= 3.939, p=0.0014.<br>**GFP TPA 200nM Versus NGF-EPE TPA 200 nM**: T_{36}= 4.249, p=0.0006.

			**GFP NT Versus BDNF-EPE NT**: T_{36}= 3.035, p=0.0177.<br><br>**GFP NT Versus NGF-EPE NT**: T_{36}= 3.346, p=0.0077.
3B	GFP: 3<br><br>BDNF-EPE: 6<br><br>NGF- EPE: 6	One-way ANOVA:<br><br>$F_{(5, 20)}$ = 10.28, p=0.0007.	**GFP TPA 200nM Versus BDNF-EPE TPA 200 nM**: T_{36}= 15.69, p<0.0001.<br><br>**GFP TPA 200nM Versus NGF-EPE TPA 200 nM**: T_{36}= 13.8, p<0.0001.<br><br>**GFP NT Versus BDNF-EPE NT**: T_{36}= 5.75, p<0.0001.<br><br>**GFP NT Versus NGF-EPE NT**: T_{36}= 6.363, p<0.0001.<br><br>**GFP NT Versus GFP TPA 200nM**: T_{36}= 6.157, p<0.0001
3D	GFP: 4<br><br>BDNF-EPE: 5<br><br>NGF- EPE: 5	One-way ANOVA:<br><br>$F_{(5, 22)}$ = 10.28, p<0.0001.	**GFP TPA 200nM Versus BDNF-EPE TPA 200 nM**: T_{22}= 2.929, p=0.0231.<br><br>**GFP NT Versus GFP TPA 200nM**: T_{22}= 5.194, p<0.0001.
3F	GFP: 3<br><br>BDNF-EPE: 6<br><br>NGF- EPE: 6	One-way ANOVA:<br><br>$F_{(5, 24)}$ = 26.62, p<0.0001.	**GFP TPA 200nM Versus BDNF-EPE TPA 200 nM**: T_{24}= 2.95, p=0.0208.<br><br>**GFP TPA 200nM Versus NGF-EPE TPA 200 nM**: T_{24}= 1.72, n.s.<br><br>**GFP NT Versus GFP TPA 200nM**: T_{24}= 6.473, p<0.0001.
3G	GFP: 3<br><br>BDNF-EPE: 6<br><br>NGF- EPE: 6	One-way ANOVA:<br><br>$F_{(5, 24)}$ = 221.1, p<0.0001.	**GFP TPA 200nM Versus BDNF-EPE TPA 200 nM**: T_{24}= 10.87, p<0.0001.<br><br>**GFP TPA 200nM Versus NGF-EPE TPA 200 nM**: T_{24}= 8.64, p<0.0001<br><br>**GFP NT Versus GFP TPA 200nM**: T_{24}= 20.79, p<0.0001.

Table S3. Detailed statistical analysis, related to Figure 4

**Figure 4**	**Biological Repetitions**	**Statistics and Post hoc test**
4B	GFP: 10 mice<br><br>BDNF-EPE: 9 mice<br><br>NGF- EPE: 11 mice	Two-way ANOVA $F_{(3, 81)}$ = 76.24, p<0.0001; Dunnett's multiple comparisons tests:<br><br>**<u>BDNF-EPE</u>**<br><br>0-120s Versus 240-300s: p<0.0001<br><br>**<u>NGF-EPE</u>**<br><br>0-120s Versus 240-300s: p<0.0001<br><br>**<u>GFP Control</u>**<br><br>0-120s Versus 240-300s: p<0.0001

4C	GFP: 10 mice<br>BDNF-EPE: 9 mice<br>NGF- EPE: 11 mice	Two-way ANOVA $F_{(10, 135)}$ = 1.891, p=0.0514; Tukey's multiple comparisons tests:<br>Extinction Day 1<br>**GFP control Versus BDNF-EPE**: p=0.0003<br>**NGF-EPE Versus BDNF-EPE**: p=0.0002<br>Extinction Day 2<br>**GFP control Versus BDNF-EPE**: p<0.0001<br>**NGF-EPE Versus BDNF-EPE**: p=0.0006<br>Extinction Day 3<br>**GFP control Versus BDNF-EPE**: p<0.0001<br>**NGF-EPE Versus BDNF-EPE**: p<0.0001<br>Extinction Day 4<br>**GFP control Versus BDNF-EPE**: p=0.0012<br>**NGF-EPE Versus BDNF-EPE**: p=0.0015<br>Extinction Day 5<br>**GFP control Versus BDNF-EPE**: p<0.0001<br>**NGF-EPE Versus BDNF-EPE**: p=0.0056
4C	GFP: 10 mice<br>BDNF-EPE: 9 mice<br>NGF- EPE: 11 mice	Two-way ANOVA $F_{(5, 135)}$ = 48.42, P<0.0001; Dunnett's multiple comparisons test:<br>**BDNF-EPE**<br>Memory test versus extinction day 1: p=0.9998<br>Memory test versus extinction day 2: p=0.3479<br>Memory test versus extinction day 3: p=0.1051<br>Memory test versus extinction day 4: p<0.0001<br>Memory test versus extinction day 5**:** p<0.0001<br>**NGF-EPE**<br>Memory test versus extinction day 1: p=0.1506<br>Memory test versus extinction day 2: p=0.0021<br>Memory test versus extinction day 3: p<0.0001<br>Memory test versus extinction day 4: p<0.0001<br>Memory test versus extinction day 5: p<0.0001<br>**GFP Control**<br>Memory test versus extinction day 1: p=0.0443

		Memory test versus extinction day 2: p<0.0001<br>Memory test versus extinction day 3: p<0.0001<br>Memory test versus extinction day 4: p<0.0001<br>Memory test versus extinction day 5: p<0.0001

Table S4. Detailed statistical analysis, related to Figure 5

**Figure 5**	**Biological Repetitions**	**Statistics**	**Post hoc test (**Dunnett's multiple comparisons test)
5A	GFP: 10 mice<br>BDNF-EPE: 9 mice<br>NGF- EPE: 11 mice	One-way ANOVA: model (all groups together): $F_{(2, 27)} = 9.487$, p=0.0008.	**GFP Control versus BDNF-EPE:** p=0.0010<br>**GFP Control versus NGF-EPE:** p=0.9179
5B	GFP: 10 mice<br>BDNF-EPE: 9 mice<br>NGF- EPE: 11 mice	One-way ANOVA: model (all groups together): $F_{(2, 27)} = 5.436$, p=0.0104.	**GFP Control versus BDNF-EPE:** p=0.0273<br>**GFP Control versus NGF-EPE:** p=0.8635
5C	GFP: 10 mice<br>BDNF-EPE: 9 mice<br>NGF- EPE: 11 mice	One-way ANOVA: model (all groups together): $F_{(2, 27)} = 3.68$, p=0.0386.	**GFP Control versus BDNF-EPE:** p=0.0261<br>**GFP Control versus NGF-EPE:** p=0.6964
5D	GFP: 10 mice<br>BDNF-EPE: 9 mice<br>NGF- EPE: 11 mice	One-way ANOVA: model (all groups together): $F_{(2, 27)} = 5.554$, p=0.0095.	**GFP Control versus BDNF-EPE:** p=0.0394<br>**GFP Control versus NGF-EPE:** p=0.6770
5E	GFP: 10 mice<br>BDNF-EPE: 9 mice<br>NGF- EPE: 11 mice	One-way ANOVA: model (all groups together): $F_{(2, 27)} = 6.524$, p=0.0049.	**GFP Control versus BDNF-EPE:** p=0.0131<br>**GFP Control versus NGF-EPE:** p=0.8979

Table S5. Detailed statistical analysis, related to Figure 6

**Figure 6**	**Number of mice**	**Statistics**	**Post hoc test (**Tukey's multiple comparisons test**)**
6B	GFP: 19<br>BDNF-EPE: 16	Mixed-effects analysis<br>Time X Treatment:<br>$F_{(14, 274)} = 5.043$, p<0.0001	<u>Extinction Day 1</u><br>**GFP controls Versus BDNF-EPE**: p=0.9940.<br><u>Extinction Day 2</u><br>**GFP controls Versus BDNF-EPE**: p=0.9711.<br><u>Extinction Day 3</u>

			**GFP control Versus BDNF-EPE**: p=0.2094.<br>Extinction Day 4<br>**GFP control Versus BDNF-EPE**: p=0.0655.<br>Extinction Day 5<br>**GFP control Versus BDNF-EPE**: p<0.0001.<br>Extinction Day 6<br>**GFP control Versus BDNF-EPE**: p=0.6592.<br>Extinction Day 7<br>**GFP control Versus BDNF-EPE**: p<0.0001.<br>RI<br>**GFP control Versus BDNF-EPE**: p=0.7991.
